# Surveillance of 3′ Noncoding Transcripts Requires FIERY1 and XRN3 in *Arabidopsis*

**DOI:** 10.1534/g3.111.001362

**Published:** 2012-04-01

**Authors:** Yukio Kurihara, Robert J. Schmitz, Joseph R. Nery, Matthew D. Schultz, Emiko Okubo-Kurihara, Taeko Morosawa, Maho Tanaka, Tetsuro Toyoda, Motoaki Seki, Joseph R. Ecker

**Affiliations:** * Plant Biology Laboratory; † Genomic Analysis Laboratory, and; ** Howard Hughes Medical Institute, Salk Institute for Biological Studies, La Jolla, California 92037, and; ‡ RIKEN Plant Science Center; § Bioinformatics and Systems Engineering Division, RIKEN Yokohama Institute, Yokohama, Kanagawa 230-0045, Japan

**Keywords:** FRY1, XRN3, genome-wide, *Arabidopsis*, methylation

## Abstract

Eukaryotes possess several RNA surveillance mechanisms that prevent undesirable aberrant RNAs from accumulating. *Arabidopsis* XRN2, XRN3, and XRN4 are three orthologs of the yeast 5′-to-3′ exoribonuclease, Rat1/Xrn2, that function in multiple RNA decay pathways. XRN activity is maintained by FIERY1 (FRY1), which converts the XRN inhibitor, adenosine 3′, 5′-bisphosphate (PAP), into 5′AMP. To identify the roles of XRNs and FRY1 in suppression of non-coding RNAs, strand-specific genome-wide tiling arrays and deep strand-specific RNA-Seq analyses were carried out in *fry1* and *xrn* single and double mutants. In *fry1-6*, about 2000 new transcripts were identified that extended the 3′ end of specific mRNAs; many of these were also observed in genotypes that possess the *xrn3-3* mutation, a partial loss-of-function allele. Mutations in *XRN2* and *XRN4* in combination with *xrn3-3* revealed only a minor effect on 3′ extensions, indicating that these genes may be partially redundant with *XRN3*. We also observed the accumulation of 3′ remnants of many DCL1-processed microRNA (miRNA) precursors in *fry1-6* and *xrn3-3*. These findings suggest that XRN3, in combination with FRY1, is required to prevent the accumulation of 3′ extensions that arise from thousands of mRNA and miRNA precursor transcripts.

During developmental transitions and environmental fluctuations, unnecessary RNAs are destined for degradation, and new functional RNAs are transcribed. Several RNA turnover pathways are involved in controlling gene expression profiles, which is important to maintain normal cells and tissues (Houseley and Tollervey 2009). Generally, mRNA decay starts by deadenylation of the 3′ poly(A) tail and then by decapping of the 5′ cap structure, followed by degradation in the 5′-to-3′ and/or 3′-to-5′ directions by exoribonucleases (Chiba and Green 2009).

In yeast, cytoplasmic Xrn1 and nuclear Rat1 are particularly prominent 5′-to-3′ exoribonucleases. Xrn1 catalyzes 5′-to-3′ mRNA degradation in multiple decay pathways, whereas Rat1 functions in the processing of ribosomal RNAs (rRNA) and small nucleolar RNAs (Garneau *et al.* 2007; Houseley and Tollervey 2009). The *Arabidopsis thaliana* genome contains three *XRN* genes named XRN2, XRN3, and XRN4, which are structurally similar to Rat1 in yeast (Kastenmayer and Green 2000). XRN2 and XRN3 are localized in the nucleus, whereas XRN4 is localized in the cytoplasm. XRN4 not only acts as an mRNA-degrading enzyme similar to the yeast Xrn1 enzyme but also acts to degrade the 3′ products that result from microRNA (miRNA)-mediated cleavage of target mRNAs (Souret *et al.* 2004; Gy *et al.* 2007; Gregory *et al.* 2008; Rymarquis *et al.* 2011). XRN4, also referred to as ETHYLENE INSENSITIVE 5 (EIN5), is required for proper ethylene signaling. It functions by directly or indirectly promoting the degradation of mRNAs of two F-box proteins that mediate protein degradation of ETHYLENE INSENSITIVE3 (EIN3), a transcription factor that elicits the ethylene response (Roman *et al.* 1995; Olmedo *et al.* 2006; Gregory *et al.* 2008). XRN2 is required for primary cleavage of pre-ribosomal RNAs and redundantly acts with XRN3 in pre-ribosomal RNA processing (Zakrzewska-Placzek *et al.* 2010). In addition to the respective functions of each family member, all XRN proteins redundantly act as endogenous RNA silencing suppressors, probably through eliminating the free 5′ ends of single-stranded RNA templates that can be recognized by RNA-dependent RNA polymerases (Gazzani *et al.* 2004; Gy *et al.* 2007). Although XRN3 has limited roles in cleavage of pre-ribosomal RNAs, its primary role in RNA processing has yet to be determined.

*FIERY1* (*FRY1*), which is an ortholog of *HAL2* from yeast, was first identified as a negative regulator of gene expression during stress responses (Xiong *et al.* 2001). This gene family encodes a 3′(2′),5′-bisphosphate nucleotidase that catalyzes 3′-phosphoadenosine 5′-phosphate (PAP), a product of sulfur assimilation, into 5′AMP and Pi (Dichtl *et al.* 1997; Gil-Mascarell *et al.* 1999; Gy *et al.* 2007). FRY1 was identified as an endogenous RNA silencing suppressor similar to the XRN gene family, because PAP is a strong inhibitor of XRN enzymatic activity (Gy *et al.* 2007). Therefore, repression of FRY1 activity leads to dysfunction of all XRN proteins through PAP overaccumulation. This effect also causes accumulation of looped RNA molecules derived from miR164b and miR168a precursors in *fry1-6* as well as slight accumulation in *xrn2xrn3* double mutants (Gy *et al.* 2007). Moreover, *fry1* mutants show severe developmental defects, such as altered root architecture, reduced growth, late flowering, and an ethylene-insensitive phenotype likely due to inhibition of XRN4/EIN5 activity (Gy *et al.* 2007; Kim *et al.* 2009; Olmedo *et al.* 2006; Chen and Xiong 2010). *fry1* mutants also exhibit drought resistance, which can be mimicked by the *xrn2xrn3xrn4* triple mutant (Hirsch *et al.* 2011).

Several recent reports revealed that numerous long non-coding RNAs, including intergenic and antisense transcripts, are abundant in the transcriptomes of many organisms, including *Arabidopsis thaliana* (Yamada *et al.* 2003; Luica and Dean 2011). Some of these transcripts possess important developmental functions through gene regulation by way of chromatin modifications. For example, a non-coding RNA arises from the antisense strand of *FLOWERING LOCUS C* (*FLC*), a major repressor of the floral transition (Liu *et al.* 2010). This antisense transcript uses two proximal and distal polyadenylation sites that are controlled by two RNA binding proteins, FCA and FPA, which in turn promote polyadenylation specifically at the proximal site (Liu *et al.* 2007; Hornyik *et al.* 2010). The antisense transcript that is adenylated at the proximal site triggers histone 3 lysine 4 demethylation and transcriptional deactivation of *FLC*, thereby inducing the transition to floral development.

However, many non-coding RNAs can be recognized by RNA surveillance mechanisms and eliminated from cells (Chekanova *et al.* 2007; Kurihara *et al.* 2009). One such RNA surveillance mechanism is nonsense-mediated decay (NMD), which fundamentally eliminates aberrant mRNAs with premature termination codons or relatively long 3′ UTRs (Maquat 2004). *UP-FRAMESHIFT* (*UPF*) genes (*UPF1*, *UPF2*, and *UPF3*) are essential for normal NMD and are evolutionarily conserved in eukaryotic organisms (Behm-Ansmant *et al.* 2007). Previous reports using genome-wide tiling arrays showed that many of the mRNA-like non-coding RNAs, including antisense transcripts, overaccumulate in *Arabidopsis upf1* and *upf3* knockdown mutants. This is likely due to the long 3′ UTRs that many of these mRNA-like non-coding RNAs possess downstream of short ORFs, which do not encode proteins and can act as a trigger for NMD (Kurihara *et al.* 2009). These results also reveal that NMD eliminates non-coding RNAs as well as aberrant mRNAs.

The exosome, a 3′-to-5′ exribonuclease complex, also plays a principal role in eliminating non-coding RNAs. Previous genome-wide tiling array analysis using inducible RNAi mutants of *RRP4* and *RRP41*, genes that encode for components of the exosome core, detected not only accumulation of hundreds of mRNAs, 5′ remnants of miRNA precursors, and several classes of structural RNAs (such as small nuclear RNAs, small nucleolar RNAs, and transfer RNAs) but also accumulation of large classes of uncharacterized non-coding RNAs (Chekanova *et al.* 2007). Many of these RNAs are transcribed from repetitive elements and siRNA-generating loci of which genomic DNA is often highly methylated, indicating a close relationship between exosome-mediated RNA decay and DNA methylation via siRNAs. The other non-coding RNAs that accumulate in *rrp4* and *rrp41* RNAi mutants include aberrant transcripts that originate from the 5′ ends (first exon) of protein-coding genes. Thus, the exosome acts as a quality control of several kinds of non-coding RNAs. Studies of the exosome and NMD have uncovered hidden layers of eukaryotic transcriptomes that comprise many classes of non-coding RNAs as well as canonical transcripts.

As described above, elimination of non-coding RNAs is often an essential mechanism for maintenance of gene expression. Here, we investigated the roles of the FRY1- and XRN-mediated non-coding RNA regulation using both whole-genome tiling arrays and next-generation RNA-sequencing (RNA-Seq) methods. In *fry1*, we detected the accumulation of several thousand non-coding transcripts that mapped to the 3′ ends of genes. We also identified accumulation of many of these same transcripts in *xrn3*, but we did not detect significantly more accumulation in *xrn2xrn3* and *xrn3xrn4* double mutants than in *xrn3*, indicating that the main activity of XRN3 is to eliminate these non-coding transcripts. Additionally, we detected accumulation of the 3′ remnants of miRNA precursors in *fry1* and *xrn3*. Therefore, we suggest that FRY1 and XRN3 are required to prevent spurious accumulation of non-coding transcripts from the 3′ end of genes.

## Materials and Methods

### Plant materials

All the wild-type (WT) and mutant *Arabidopsis* plants used in this study are from the Columbia (Col-0) background. *fry1-6* (Salk_020882), *xrn2-1* (Salk_041148), *xrn3-3* (Sail_1172C07), *xrn4-6* (Salk_014209), *xrn2-1xrn3-3*, *xrn2-1xrn4-6*, and *xrn3-3xrn4-6* were described previously (Gy *et al.* 2007). *xrn2-4* (Salk_073255) was recovered from the Salk T-DNA insertion collection [http://signal.salk.edu/cgi-bin/tdnaexpress and Alonso *et al.* (2003)].

### Tiling array analysis

The GeneChip *Arabidopsis* tiling array set (1.0F Array and 1.0R Array; Affymetrix, Santa Clara, CA) was used (Zhang *et al.* 2006). Plant growth conditions, RNA extraction, probe synthesis, and array hybridization were carried out as described previously (Matsui *et al.* 2008; Kurihara *et al.* 2009). The ARTADE-based method (P-initial < 10^−8^) was used to predict novel (unannotated) transcripts from the expression data (ARTADE v1.2.1.1) (Toyoda and Shinozaki 2005). The *Arabidopsis* genome annotation used in tiling array analysis was based on the TAIR8 (ftp://ftp.arabidopsis.org/home/tair/Genes/TAIR8_genome_release/). The transcripts predominantly upregulated in the mutants were identified by the Mann-Whitney *U*-test (FDR α = 0.05).

### RNA-Seq analysis

Wild-type, *fry1-6*, *xrn3-3*, *xrn2-1xrn4-6*, and *xrn3-3xrn4-6* were grown in Linsmaier and Skoog (LS; Caisson Laboratories, North Logan, UT) media containing 1% sucrose and 0.85% agar at 23° under conditions of 16 hr of light and 8 hr of darkness for 2 weeks. Wild-type (WT3w) and *xrn2-1xrn3-3* were grown in soil (Metro Mix 250; Grace-Sierra, Boca Raton, FL) at 23° under conditions of 16 hr of light and 8 hr of darkness for 3 weeks. Total RNA was extracted from the plants using Trizol reagent (Invitrogen, Carlsbad, CA). Poly(A)^+^ fraction was separated from 80 μg of total RNA using Oligotex mRNA Mini Kit (Qiagen, Valencia, CA) and fragmented using Fragmentation Reagents (Applied Biosystems/Ambion, Austin, TX) at 70° for 15 min. Fifty nanograms of the fragmented RNA was used to generate strand-specific RNA-Seq library according to the Directional mRNA-Seq Library Preparation Protocol (Illumina). RNA-Seq libraries were sequenced for 42 cycles using the Illumina Genome Analyzer IIx (WT and *fry1-6*) or for 50 cycles using the HiSeq 2000 (*xrn3-3*, *xrn3-3xrn4-6*, 3-week-old WT and *xrn2-1xrn3-3*) according to manufacturer’s instruction. Image analysis and base calling were performed with the standard Illumina pipeline. Read sequences were aligned with the Tophat software to the TAIR9 reference genome (Trapnell *et al.* 2009). Reads that aligned to multiple positions were discarded. Reads per kilobase of transcript per million (RPKM) values were calculated using the Refiner Genome module developed by Genedata Expressionist (Genedata Inc., Lexington, MA). The *Arabidopsis* genome annotation used was based on the TAIR9 (ftp://ftp.arabidopsis.org/home/tair/Genes/TAIR9_genome_release/). The transcripts predominantly upregulated in the mutants were identified by using a Student *t*-test (P-value < 0.1).

### MethylC-Seq analysis

Wild-type and *fry1-6* were grown in LS (Caisson Laboratories) media containing 1% sucrose and 0.85% agar at 23° under conditions of 16 hr of light and 8 hr of darkness for 2 weeks. One microgram of gDNA was isolated from this tissue using the Qiagen Plant DNeasy Kit (Qiagen). DNA was sheared to ∼100 bp using the Covaris S2 System using the following parameters: cycle number = 6, duty cycle = 20%, intensity = 5, cycles/burst = 200, and time = 60 sec. Sonicated DNA was purified with a mini-elute column (Qiagen). Sequencing libraries were constructed using the NEBNext DNA Sample Prep Reagent Set 1 (New England Biolabs, Ipswich, MA) following the manufacturer’s instructions, except for the following modifications. Methylated adapters were used instead of the standard genomic DNA adapters from Illumina. Ligations were purified with AMPure XP beads (Beckman, Brea, CA). A total of 450 ng of DNA was bisulfite treated using the MethylCode Kit (Invitrogen) according to the manufacturer’s instructions and then PCR amplified using Pfu Cx Turbo (Agilent, Santa Clara, CA) instead of using the Phusion Taq included in the NEBNext kit using the following PCR conditions (2 min at 95°, 4 cycles of 15 sec at 98°, 30 sec at 60°, 4 min at 72°, and 10 min at 72°).

### Analysis of differentially methylated regions

DMRs were identified using the methylPipe package in R (Lister *et al.* 2011; Pelizzola, unpublished results). Each specific methylation context (CG, CHG, and CHH) was scanned genome-wide requiring at least 10 mC differences within a 100 bp window. The methylation level of the sites within a window was then compared across all samples using a using a Kruskal-Wallis test. Next, these potential DMRs were consolidated by joining neighboring DMRs that occur within 100 bp of each other. The P-values of joined DMRs were combined using Fisher’s Method. The P-values of these joined DMRs were then adjusted for multiple hypotheses testing with the Benjamini-Hochburg method as implemented in R, and any DMR with an adjusted P-value below 0.01 was retained. Furthermore, a stringent requirement of an 8-fold difference in methylation density between the least methylated and most methylated sample was required.

### Northern blot analysis

Fifteen micrograms of total RNA were loaded into 1% denaturing agarose gel containing 18% formaldehyde and MOPS buffer (0.2 M MOPS, 80 mM Na-acetate, and 10 mM EDTA, pH 7.0), subjected to electrophoresis at 100 V in MOPS buffer, and transferred onto Hybond N+ membrane (GE Healthcare, Piscataway, NJ) by capillary blotting method in 20 × SSC buffer (3 M sodium chloride, 300 mM trisodium citrate, pH 7.0), followed by UV-crosslinking with 120,000 μJ/cm^2^. Prehybridization and hybridization with each specific probe were performed with the PerfectHyb hybridization buffer (Sigma-Aldrich, St. Louis, MO) at 65° for 1 hr and overnight, respectively. The membrane was washed in the first wash buffer (2 × SSC, 0.1% SDS) for 10 min twice and then in the second wash buffer (0.5 × SSC, 0.1% SDS) for 20 min three times. The membrane was exposed to Imaging Plate BAS-MS2040 for 2 hr and the signals were detected using Bio-Image analyzer BAS-2500 (Fujifilm, Tokyo, Japan).

DNA probes were constructed by random priming reactions with a ^32^P-dCTP using the Megaprime DNA-labeling system (GE Healthcare) according to manufacturer’s instruction. DNA fragments used for these reactions were PCR amplified from genomic DNA using the following primer sets: mRNA_F and mRNA_R for mRNA detection, 3′ext_F and 3′ext_R for 3′ extension detection, and TUB8_F and TUB8_R for *TUB8* mRNA detection (supporting information, Table S1).

### Self-ligation–mediated RT-PCR

Total RNA was extracted using Trizol reagent (Invitrogen) from 2-week-old seedlings grown in LS media as described above. Poly(A)^+^ RNA was separated from total RNA using Poly(A) Purist kit (Applied Biosystems/Ambion) according to the manufacturer’s instructions. The RNA (500 ng) was treated with or without calf intestinal phosphatase (CIP, 10 U; New England Biolabs, Cambridge, MA) in a 20 μL volume and then tobacco acid pyrophosphatase (TAP, 2 U; Epicentre Biotechnologies, Madison, WI) in a 30 μL volume. The modified or unmodified RNA molecules were self-ligated using T4 RNA ligase (20 U; Promega) in a 50 μL volume, followed by reverse-transcription using SuperScript II Reverse Transcriptase and Oligo dT primer in a 20 μL volume. As a PCR template, 0.25 μL of RT product was used. The PCR was carried out in a 20 μL volume using Phire Hot Start DNA polymerase (New England Biolabs) and the following primer set (circle_F and circle_R; Table S1). The amplification program was 35 cycles of 5 sec at 98°, 15 sec at 55°, and 20 sec at 72°. The resulting PCR product was loaded into a 1.5% agarose gel, subjected to electrophoresis in TBE (50 mM Tris-HCl, 48.5 mM boric acid, 2 mM EDTA, pH 8.0) buffer at 100 V and visualized under UV light.

### Quantitative RT-PCR analysis

Total RNA was extracted from 20-day-old seedlings grown in LS media containing 1% sucrose and 0.85% agar at 23° under conditions of 16 hr of light and 8 hr of darkness. Ten micrograms of total RNA were digested in 50 μL of TURBO DNase (Applied Biosystems/Ambion) for 30 min and recovered by acidic phenol:chloroform extractions and ethanol precipitation. Two micrograms of the DNase-treated RNA was used for the reverse-transcription reaction in 20 μL using the SuperScript II reverse transcriptase (Invitrogen) and Oligo dT primer according to the manufacturer’s instructions. As a template for PCR amplification, 0.25 μL of the RT product was used. The PCR was carried out in 20 μL using Taq DNA polymerase for amplification of mRNAs and 3′ extension of At1g16410, or using Phire Hot Start DNA polymerase (New England Biolabs) for amplification of 3′ extensions (with the exception of At1g16410), pri-miRNA_5′s, and pri-miRNA_3′s. Primer sets used here and cycle numbers of PCR are listed in Table S1.

### Accession numbers

Raw data from tiling arrays and RNA-Seq were deposited in Gene Expression Omnibus under the accession numbers GSE32977 and GSE34654, respectively. Raw data from MethylC-Seq were deposited in the Sequence Read Archive (SRA) of NCBI under accession number SRA049100.

## Results

### Identification of novel transcripts extending from the 3′ ends of mRNAs

**Figure 1:**
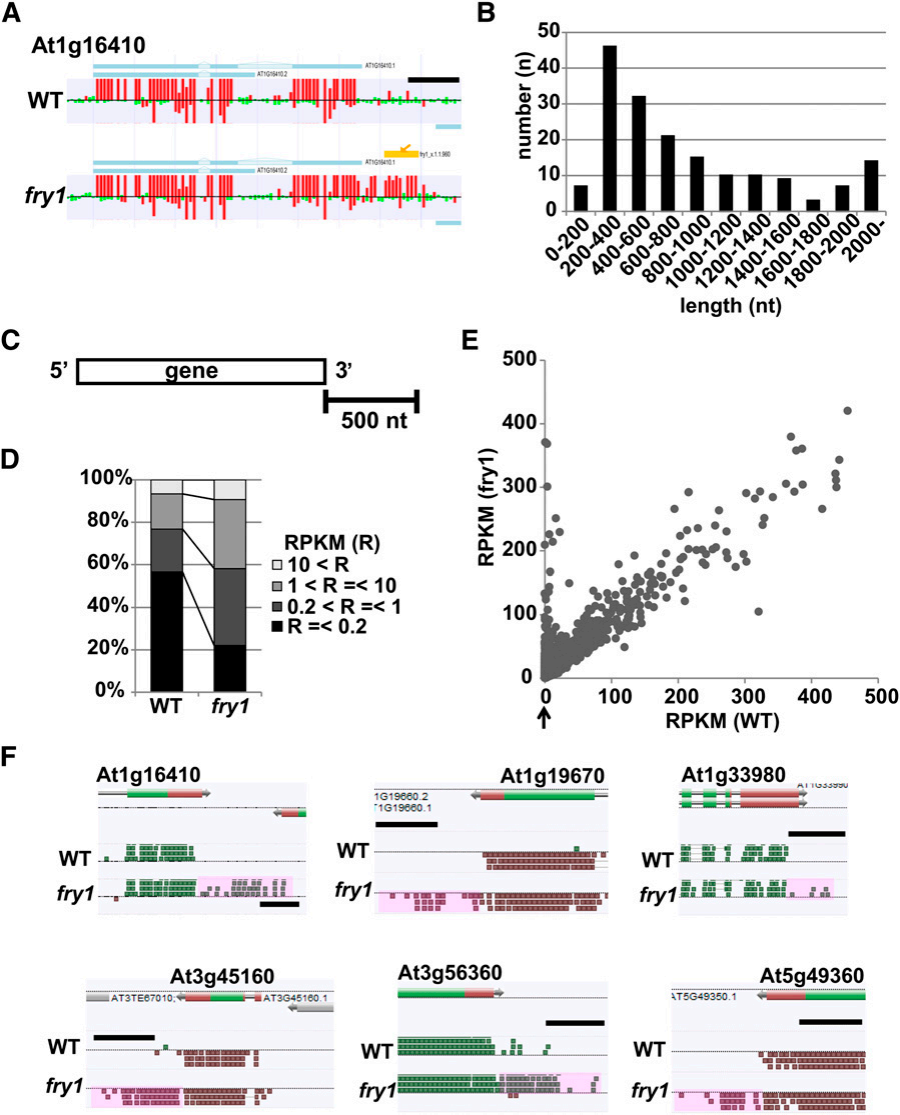
Identification of novel transcripts extending the 3′ ends of mRNAs. (A) Example (At1g16410 locus) of novel 3′ extended signals (3′ extensions) identified by the ARTADE program (Toyoda and Shinozaki 2005) in tiling array data from *fry1-6*. Vertical red bars indicate signal intensities from each probe. Horizontal blue and light blue regions are exons and introns, respectively, in the gene model of At1g16410. The horizontal orange region indicated by the arrow is a predicted 3′ extension. The length of black scale bar is 500 nt. (B) Distribution of estimated lengths of 171 3′ extensions identified in the tiling array analysis of *fry1-6*. (C) A schematic of the 500 nt downstream region used for calculation of RPKM values. (D) Comparison of accumulation profiles in the range from the 3′ ends of genes to 500 nt downstream between wild-type and *fry1-6* (RNA-Seq). RPKM values of all expressed transcripts (total 19,104) in wild-type or *fry1-6* were classified into four categories. (E) A scatter plot of average RPKM values between wild-type and *fry1-6*. The 3′ regions with RPKM values less than 500 were plotted. (F) Examples of 3′ extensions identified in *fry1-6* by filtering through four parameters [Fold (*fry1-6*/WT) > 5, RPKM average (*fry1*) > 1, P-value < 0.1, non-pri-miRNA]. Pink regions are 3′ extensions. The length of the black scale bar is 500 nt.

Strand-specific whole-genome tiling array analysis of *fry1-6* and *xrn* single mutants (*xrn2-4*, *xrn3-3*, *xrn4-6*) was performed to identify the roles of XRNs and FRY1 in transcript accumulation. The *xrn2-4* allele (Salk_073255) has a T-DNA insertion in the 12^th^ intron, resulting in extremely reduced mRNA expression (see Figure S1A). These data were normalized together with previous tiling array data generated for *upf* mutants (Kurihara *et al.* 2009) to reduce misidentification of unannotated transcripts, such as non-coding RNAs. The ARTADE program, which predicts novel transcripts *de novo* (Toyoda and Shinozaki 2005) uncovered 171 novel transcripts located less than 300 nucleotides (nt) downstream of the 3′ ends of upstream mRNAs in *fry1-6* [Fold (*fry1-6*/WT) > 2, P-initial < 10^−8^, FDR α = 0.05] (Figure 1A and Table S2). Henceforth, we will refer to these novel transcripts as 3′ extensions. Interestingly, 61% (104) of the estimated lengths of the 3′ extensions were less than 800 nt (Figure 1B). The results from the tiling array analyses can be observed on OmicBrowse at http://omicspace.riken.jp/gps/group/psca7.

We suspected that many low abundance 3′ transcript extensions may not be detected using tiling array analysis. To overcome this limitation, strand-specific RNA-Seq was carried out using an Illumina Genome Analyzer IIx to investigate these RNA populations at single base resolution. Unlike the tiling array technique, the RNA-Seq method does not suffer from background noise (cross-hybridization of RNAs with multiple probes) because the vast majority of reads can be uniquely mapped in the *Arabidopsis* reference genome (Lister *et al.* 2008). First, three biological replicates of RNA-Seq were carried out using mRNA prepared using wild-type and *fry1-6* seedlings. We identified 33.2 and 35.0 million sequenced reads that uniquely aligned to the TAIR9 reference genome sequence in wild-type and *fry1-6*, respectively. For quantification of transcript levels, RPKM values were calculated for the regions extending 500 nt downstream from the 3′ ends of all annotated genes. (Figure 1C). This range was selected because the lengths of most of 3′ extensions observed from previous tiling array analysis were less than 800 nt in length (Figure 1B). RPKM values of these 3′ regions tended to be higher in *fry1-6* than in wild-type (data are displayed by classifying them into four categories: RPKM ≦ 0.2. 0.2 < RPKM ≤ 1, 1 < RPKM ≤ 10, and RPKM > 10; Figure 1D). In *fry1-6*, novel transcripts were detected at the 3′ ends of 6901 genes (Figure 1E, see vertical 0 line of WT, indicated by an arrow). RNA-Seq data were then further filtered by four parameters [Fold change (*fry1-6*/WT) > 5, RPKM average (*fry1*) > 1, P-value < 0.1, non-pri-miRNA], which resulted in the identification of 2230 high-quality candidate 3′ transcript extensions (Figure 1F, Table 1, and Table S2). The RNA-Seq data can be visualized using the AnnoJ genome browser located at http://neomorph.salk.edu/dev/pages/XRN.html.

**Table 1:** Summary of 3′ extensions in RNA-Seq

Genotype	3′ Extension*^a^*	Raw*^b^*	Platform	Replicate	Total Number of Reads (in millions)
*fry1-6*	2,230	6,901	GAII	3	35.0
*xrn3-3*	528	16,570	HiSeq	2	65.7
*xrn2xrn3*	473	16,471	HiSeq	2	58.5
*xrn2xrn4*	64	16,834	HiSeq	2	60.8
*xrn3xrn4*	392	14,028	HiSeq	2	58.7

a The numbers of 3′ extension candidates were estimated by filtering RNA-Seq data by four parameters (Fold > 5, RPKM > 1, *P*-value < 0.1, non-pri-miRNA).

b This category indicates the numbers of new transcripts that have RPKM values on 500 nt downstream from the 3′ ends of genes.

**Figure 2:**
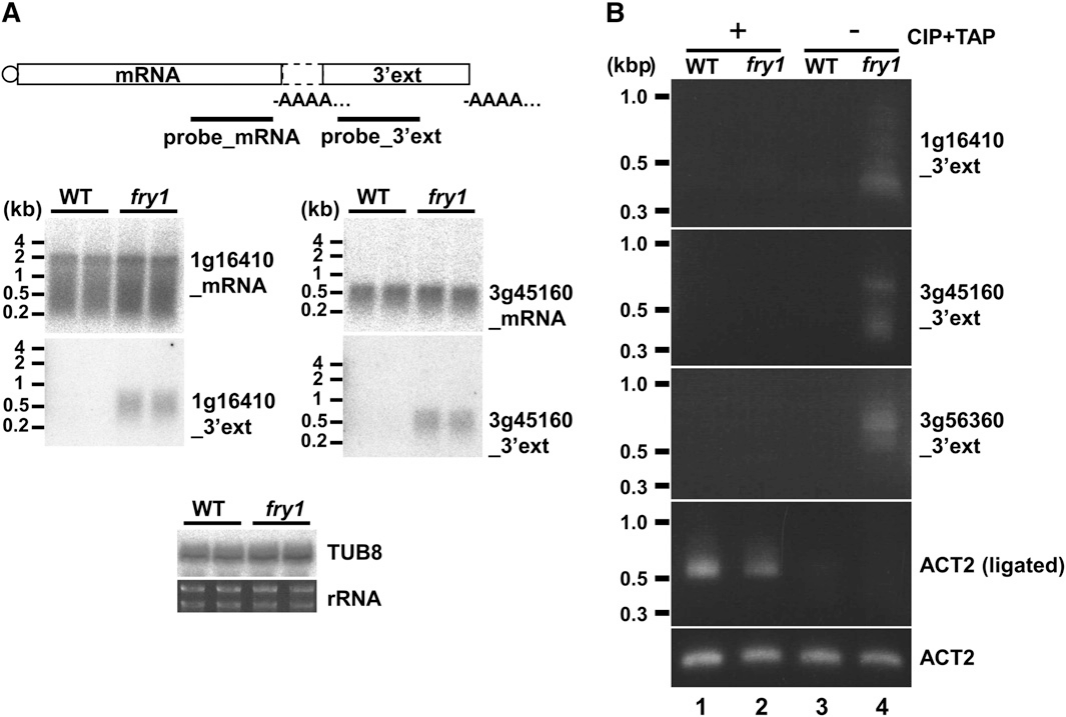
Characterization of 3′ extensions. (A) Northern blot of mRNAs and 3′ extensions of At1g16410 and At3g45160. Positions of probes used are shown in the illustration of the mRNA and the 3′ extension. Samples from two biological replicates are loaded on each blot. *TUB8* mRNA and ribosomal RNA were used as internal controls. (B) Self-ligation–mediated RT-PCR analysis. The experimental procedure is shown in Figure S1C. *ACT2* mRNA was used as an internal control. CIP, calf intestinal phosphatase; TAP, tobacco acid pyrophosphatase.

Because of the possibility that these 5′ mRNAs, which are accompanied with 3′ extensions, and the candidate 3′ extensions may, in fact, be two separate transcripts rather than one extended transcript, northern blot analysis was carried out on two genes (At1g16410 and At3g45160) using two probes covering the mRNA or its 3′ extension. We did not uncover evidence for single transcripts from either of the genes tested (Figure 2A). Furthermore, RT-PCR across the junction between each mRNA and its 3′ extension using an additional six transcript pairs did not provide evidence for gene transcript extension (Figure S1B). Therefore, we conclude that the majority of 5′ mRNAs and 3′ “extensions” are generally two separate transcripts, although we cannot rule out the possibility that low levels of single transcripts exist or that they exist for a limited number of untested loci.

Previous reports suggest that the exoribonuclease Rat1/Xrn2 degrades 3′ read-through products after endonucleolytic cleavage at the poly(A) site of these RNA polymerase II (Pol. II)–transcribed mRNAs (Kim *et al.* 2004; West *et al.* 2004). Such 3′ extensions are uncapped, which is an important signature of exoribonuclease substrates (Gregory *et al.* 2008). To examine whether the 5′ end of the 3′ extension transcripts contains a canonical 5′ cap, we performed self-ligation–mediated RT-PCR analysis for the 3′ extensions of At1g16410, At3g45160, and At3g56360 (Figure S1C). In this assay, the poly(A)-enriched RNA fraction is treated with or without CIP and TAP enzymes and then self-ligated to make a circle, followed by RT-PCR using a primer set that amplifies fragments across the gap between 5′ and 3′ ends of the RNA. Only capped RNA species can be amplified after treatment with CIP and TAP enzymes, whereas only uncapped RNA species can be amplified without CIP and TAP treatment. Specific amplification products from 3′ extensions were detected only in *fry1-6*, and not in wild-type, when RNAs were not pretreated with these enzymes (Figure 2B). The smeary bands observed in lane 4 most likely indicate that the position of 5′ and/or 3′ ends of 3′ extensions is not uniform, which is probably due to degradation and/or variable poly(A) tail and nonstop PCR amplification. On the other hand, when RNAs were pretreated with TAP and CIP, specific amplification products from *ACT2* mRNA were detected in both WT and *fry1-6*, demonstrating that the enzyme treatments were effective. These results indicate that the 3′ extensions detected in *fry1-6* mutants are very likely equivalent to Pol II 3′ read-through products.

### mRNA 3′extensions often occur in abundantly expressed transcripts

When the set of 2230 mRNAs with 3′ extensions were analyzed by gene ontology (GO) annotations (categories cellular component, molecular component, and biological component), there were few significant differences in represented GO terms between all *Arabidopsis* genes expressed in *fry1-6* and the 2230 mRNAs. The only exception was that in all three categories, the percentages of genes of unknown function were significantly lower for the 2230 mRNAs with 3′ extensions than for all genes expressed in *fry1-6* (Figure S2A). Therefore, these data suggest that FRY1 does not target specific classes of genes but, instead, is required for general surveillance that prevents these 3′ extensions.

**Figure 3:**
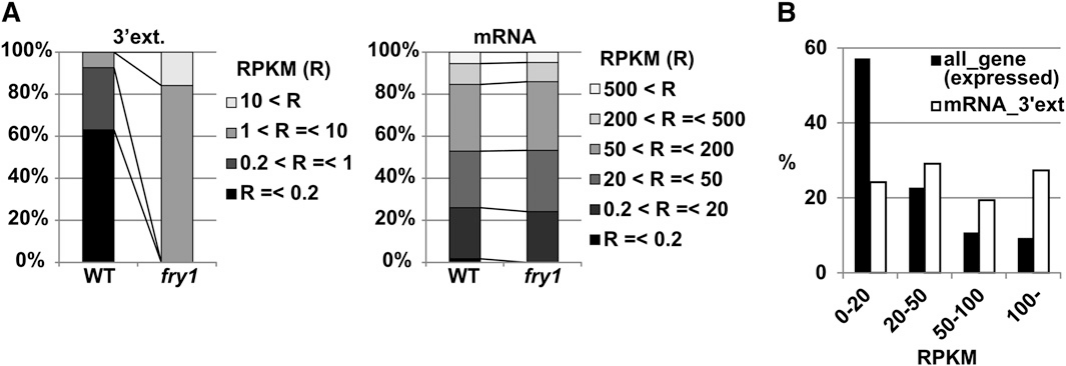
Many actively transcribed genes possess 3′ RNA extensions. Comparison of accumulation profiles of 2230 3′ extensions and the 5′ mRNAs between wild-type and *fry1-6*. RPKM values of 3′ extensions and the mRNAs were classified into four and six categories, respectively. (B) Distribution of RPKM values of mRNAs tailed with 2230 3′ extensions. The population “all_gene” comprising mRNAs with RPKM values in *fry1-6* was used for control of comparison. ext, extension.

Next, we compared the accumulation of these 2230 mRNAs and their 3′ extensions between wild-type and *fry1-6*. Although accumulation of 3′ extensions was apparently higher in *fry1-6* than in wild-type, accumulation of the 5′ mRNAs was comparable between both genotypes (Figure 3A). However, the majority of the mRNAs with 3′ extensions have relatively higher RPKM values compared with all expressed genes (Figure 3B and Figure S2B), indicating that actively transcribed genes tend to possess 3′ extensions.

### XRN3 represses 3′ extension transcripts

FRY1 is required for catalyzing conversion of the XRN inhibitor PAP into 5′AMP, thereby promoting XRN activities (Gy *et al.* 2007; Kim *et al.* 2009). In addition to the *fry1-6* mutant, slightly increased accumulation of some 3′ extensions in *xrn3-3* were observed from the tiling array analysis (Figure S3A). However, it was difficult to detect 3′ extensions in *xrn3-3*, possibly because *xrn3-3* is a knockdown allele with a T-DNA insertion in the promoter region that results in reduced expression of *XRN3* mRNA rather than in a complete knockout (Figure S1A; Gy *et al.* 2007). Therefore, the effect on accumulation of 3′ extensions might be limited due to the allele used and low detection sensitivity of the tiling array in this experiment. As knockout mutant of XRN3 gene is lethal, we could not use it here (Gy *et al.* 2007).

**Figure 4:**
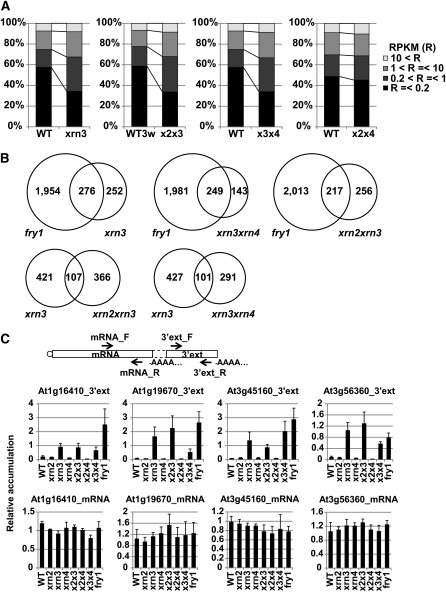
The major activity of XRN3 is to eliminate 3′ extensions. (A) Comparison of accumulation profiles in wild-type and each mutant in the range from the 3′ end of genes to 500 nt downstream. RPKM values of all expressed transcripts in wild-type or each mutant were classified into four categories. Total numbers are 18,431, 17,247, 18,480, and 15,323 in wild-type or *xrn3-3*, *xrn2xrn3*, *xrn3xrn4*, and *xrn2xrn4*, respectively. Averages RPKM values of two RNA-Seq replicates were used. Tissues use for wild-type (WT), *xrn3-3*, *xrn3xrn4*, and *xrn2xrn4* were 2-week-old plants grown on nutrient medium, whereas *xrn2xrn3* and the control wild-type (WT3w) were 3-week-old plants grown in soil. (B) Venn diagrams of the overlap between 3′ extensions identified in *fry1-6* and those identified in *xrn3-3*, *xrn2xrn3*, and *xrn3xrn4*, and the overlap between in *xrn3-3* and in *xrn3* double mutants. (C) Quantitative RT-PCR analysis of four 3′ extensions and the corresponding 5′ adjacent mRNAs. Positions of primers used are shown in the illustration of the mRNA and the 3′ extension. Vertical axes show relative accumulation normalized against *ACT2* expression. x2x3 = *xrn2xrn3*; x2x4 = *xrn2xrn4*; x3x4 = *xrn3xrn4*. Additional qRT-PCR data are presented in Figure S3C.

To increase detection sensitivity, two biological replicates of directional RNA-Seq (on the Illumina HiSeq 2000 platform) were performed using *xrn3-3* to examine whether XRN3 activity degrades the 3′ read-through products described above. Additionally, two biological replicates of RNA-Seq using *xrn2-1xrn3-3*, *xrn2-1xrn4-6*, and *xrn3-3xrn4-6* double mutants were carried out to investigate possible redundancies among the three XRN enzymes. The total numbers of sequenced reads mapped to the TAIR9 reference genome sequence for each genotype were 65.7, 58.7, 60.8, 35.7, and 58.5 million in *xrn3-3*, *xrn3xrn4*, *xrn2xrn4*, 3-week-old wild-type, and *xrn2xrn3*, respectively. Analyses of the results revealed that RPKM values of the 3′ regions composed of the 500 nt regions downstream of genes were typically greater in *xrn3-3*, *xrn2xrn3*, and *xrn3xrn4* (but not *xrn2xrn4*) when compared with wild-type (Figure 4A). When compared with *fry1-6* (Figure 1D), RPKM values were lower, which is consistent with a previous report that *xrn3-3* is a weak allele that results in fewer morphological defects than *fry1-6* (Gy *et al.* 2007).

When these RNA-Seq data were stringently filtered using four parameters (Fold change > 5, RPKM average > 1, P-value < 0.1, non-pri-miRNA), we identified 528, 473, and 392 3′ extension candidates in *xrn3*, *xrn2xrn3*, and *xrn3xrn4*, respectively (Table 1 and Table S3). These candidates significantly overlapped with the 2230 3′ extensions observed in *fry1-6* (Figure 4B). The abundance of the 2230 mRNAs with 3′ extensions identified in *fry1-6* showed little change in *xrn3*, *xrn2xrn3*, *xrn2xrn4*, and *xrn3xrn4* compared with wild-type. However, the abundance of the concomitant 2230 3′ extensions was notably increased in *xrn3*, *xrn2xrn3*, and *xrn3xrn4*, but not in *xrn2xrn4* (Figure S3B). The candidates in *xrn3-3* do not seem to widely overlap with those in *xrn3* double mutants (Figure 4B). Of the 528 candidates in *xrn3-3*, 20.3% and 19.1% overlap with those in *xrn2xrn3* and *xrn3xrn4*, respectively. However, this result is due, in part, to the limitation of threshold values used for candidate identification. For example, when alternative (less stringent) threshold values are used to identify 3′ extension candidates (Fold change > 2, RPKM average > 1, non-pri-miRNA), 44.8% and 51.5% of newly calculated candidates in *xrn3-3* overlap with those in *xrn2xrn3* and *xrn3xrn4*, respectively. These results reveal that significant redundancy in suppressing 3′ extensions was not detected between XRN3 and XRN2/XRN4, indicating the XRN3 enzyme possesses unique specificity in 3′ end processing/turnover.

Quantitative RT-PCR (qRT-PCR) analysis was used to examine the abundance of selected 5′ mRNAs and 3′ extensions in all genotypes. Two primer sets were used to amplify the 5′ mRNAs (mRNA_F and mRNA_R) and 3′ extensions (3′ext_F and 3′ext_R), respectively (Figure 4C and Table S3). These experiments revealed the accumulation of 3′ extensions was not correlated with accumulation of the 5′ mRNAs, consistent with the results of the RNA-Seq analyses (Figure 4B and Figure S3C). In this assay, we observed either compensation for reduced XRN3 activity in *xrn3-3* by XRN2/XRN4 or a dependency on the combination of mutations in the double mutants. For example, in the case of At3g45160, accumulation of the 3′ extension in *xrn3xrn4* was higher than in *xrn3-3*. By contrast, in the case of At1g19670, accumulation of the 3′ extension observed in *xrn3xrn4* was lower than in *xrn3-3*. These results suggest that XRN3 is the main component eliminating 3′ extensions and that other XRNs redundantly act to compensate, in some cases, for reduced XRN3 activity in the *xrn3-3* mutant. In this context, it is apparent that XRN3 possesses at least of one of the features that define yeast Rat1-type enzymes: involvement in termination of transcription. However, the other *Arabidopsis* Rat1 orthologs, XRN2 and XRN4, exhibit little or none of this function. Surprisingly, XRN2 does not seem to act redundantly with XRN3 in targeting of 3′ extensions for degradation, even though both XRN2 and XRN3 localize to the nucleus (Kastenmayer and Green 2000).

### Transcript 3′ extensions do not lead to DNA methylation of the genomic region of origin

**Figure 5:**
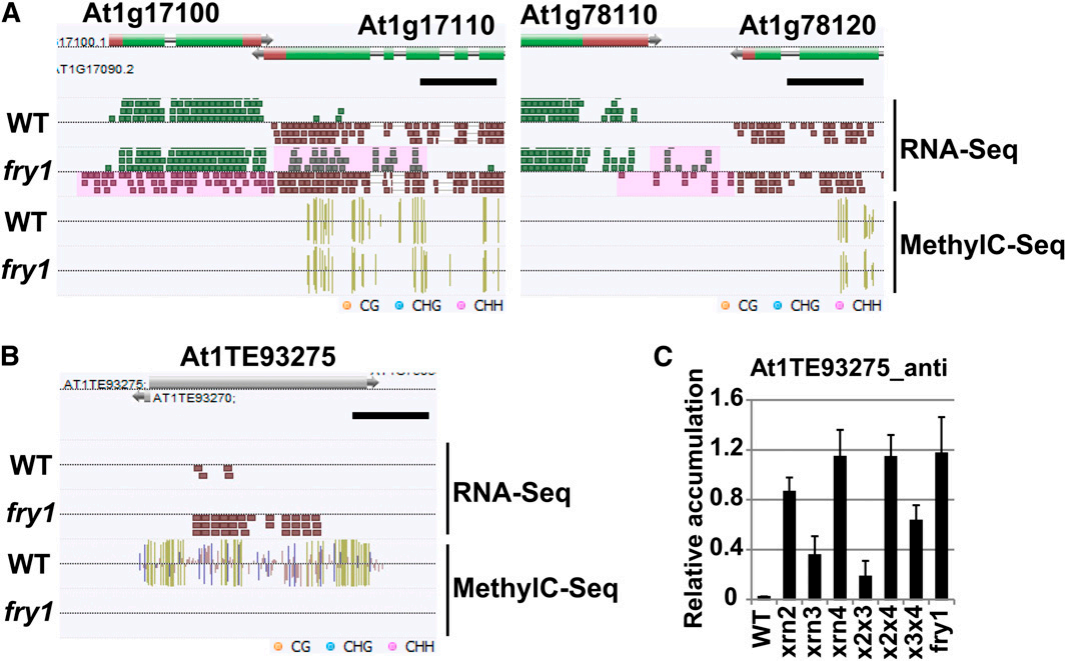
With few exceptions, 3′ extensions do not lead to *de novo* DNA methylation of the 3′ extension originating loci. (A) Examples of RNA-Seq and MethylC-Seq data from loci that exhibit overlapping transcripts. Pink regions are 3′ extensions. (B) At1TE93275 locus. The length of black scale bar is 500 nt. (C) Quantitative RT-PCR analysis of the antisense transcript of At1TE93275. Vertical axes show relative accumulation normalized against *ACT2* expression. x2x3 = *xrn2xrn3*; x2x4 = *xrn2xrn4*; x3x4 = *xrn3xrn4*.

A total of 953 3′ extension candidates potentially overlapped with flanking opposite-strand transcripts from neighboring genes while a total of 24 pairs of 3′ extension candidates were confirmed to overlap with each other (Figure 5A). We defined these 3′ extensions by postulating that their lengths are ∼500 nt. This range was used because the predicted lengths of most of 3′ extensions observed in tiling array analysis were less than 800 nt (Figure 1B). It is hypothesized that overlap between neighboring Xrn-sensitive antisense transcripts provides a source of double-stranded RNAs which may lead to pools of siRNA species that in turn could mediate *de novo* DNA methylation of these 3′ extension-generating loci (Zhang and Zhu 2011). To investigate whether these 3′ extensions correlated with *de novo* DNA methylation, we performed genome-wide MethylC-Seq analysis in *fry1-6* and wild-type (Lister *et al.* 2008; Schmitz and Zhang 2011). A total of 52.8 and 53.0 million reads were aligned to the reference genome, resulting in ∼42× coverage (∼21× per strand coverage) of both wild-type and *fry1-6*, respectively. In total, we identified 35 differentially methylated regions (DMR) in *fry1-6* compared with wild-type (P-value > 0.01, 8-fold difference in methylation levels as determined by combining levels from all three methylation contexts, CG, CHG, and CHH). However, none of the DMRs correlated with the identified 3′ extensions from our previous analysis (Figure 5A and Table S4), indicating that 3′ extensions do not lead to *de novo* DNA methylation of the 3′ extension originating loci.

A recent report using transcriptome tiling array analysis (Sonmez *et al.* 2011) revealed that expression of some 3′ downstream regions were increased in an *fcafpa* double mutant, resulting in 3′ transcript extensions similar to those reported here. Moreover, the expression of one transposon (At1TE93275) was reported to be increased, and DNA methylation at this locus was reduced in *fcafpa*. Similarly, we found that expression of antisense transcripts originating from this transposon was increased, and DNA methylation was drastically reduced in *fry1-6* (Figure 5B). However, *xrn2* and *xrn4* mutations more strongly affected the expression of antisense transcripts originating from this locus compared with *xrn3* mutations, and the combination of the *xrn3* mutation with *xrn2* or *xrn4* mutations suppressed the expression of this transcript by way of an unknown mechanism (Figure 5C and Figure S4). Therefore, the mechanism regulating expression of this transposon seems to be independent of the XRN3-controlled 3′ extension.

### FRY1 and XRN3 repress 3′ remnants of miRNA precursors

MicroRNAs (miRNAs) are regulatory small RNAs produced through DICER-LIKE1 (DCL1)–mediated cleavage of the stem-loop structure of primary miRNA precursors (pri-miRNA) (Kurihara and Watanabe 2004; Song *et al.* 2010). They execute endonucleolytic cleavage of target mRNAs with target sites complementary to the miRNAs (Chapman and Carrington 2007; Voinnet 2009). Previous reports have demonstrated that XRN4 and FRY1 cooperatively repress the accumulation of some 3′ products of miRNA-mediated cleavage of target loci (Souret *et al.* 2004; Gy *et al.* 2007; Gregory *et al.* 2008). In our tiling array and RNA-Seq analyses, we also detected overaccumulation of such 3′ products of miRNA targets in *xrn4-6* and *fry1-6* (Figure S5A). We calculated RPKM values of the previously predicted miRNA targets in two ranges: 200 nt downstream of start sites of genes, and 200 nt upstream of 3′ ends of genes in wild-type and *fry1-6*. The results showed that many of RPKM values of start sites of 165 miRNA targets were comparable, but RPKM values of their 3′ ends were higher in *fry1-6* (Figure S5B). These results indicate and support a model in which the FRY1- and XRN4-mediated decay pathway plays a major role in degrading 3′ products of miRNA-mediated cleavage of targets.

**Figure 6:**
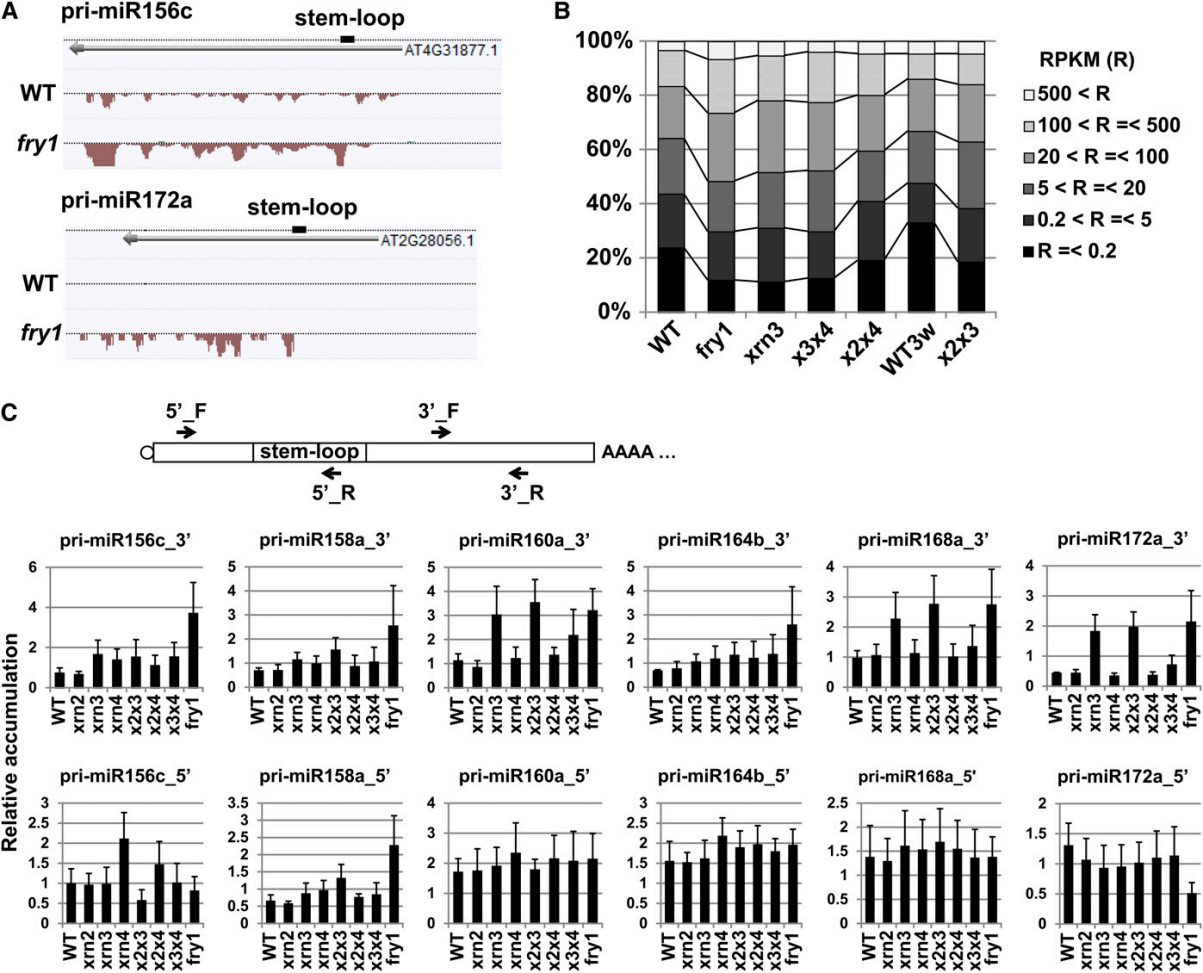
FRY1 and XRN3 repress 3′ remnants of miRNA precursors. (A) Examples of pri-miRNAs (pri-miR156c and pri-miR172a) showing increased accumulation. (B) Comparison of accumulation profiles of 151 expressed pri-miRNAs. Averages of RPKM values of three replicates of RNA-Seq in wild-type and *fry1-6* and two replicates of RNA-Seq in *xrn3-3*, *xrn3xrn4*, 3-week-old wild-type, and *xrn2xrn3* were used. (C) Quantitative RT-PCR analysis of six pri-miRNA_3′s and the pri-miRNA_5′s. Positions of primers used are shown in the illustration of pri-miRNA. Vertical axes show relative accumulation normalized against *ACT2* expression. x2x3 = *xrn2xrn3*; x2x4 = *xrn2xrn4*; x3x4 = *xrn3xrn4*.

FRY1 and three XRN exoribonucleases cooperatively repress some loop structures released after DCL1-mediated cleavage of pri-miRNAs in the miRNA processing step (Gy *et al.* 2007). Our previous report also showed that 5′ cleavage remnants of pri-miRNAs are degraded by the exosome complex (Chekanova *et al.* 2007). Our current analysis has newly revealed overaccumulation of sequenced reads that map to pri-miRNA loci in the *fry1-6* mutant compared with wild-type (Figure 6A). The enriched transcript regions are located downstream of the stem-loop structures of pri-miRNAs. As most of the pri-miRNA gene models in TAIR9 include stem-loop regions but not 3′ downstream regions adjacent to the stem-loop regions, we calculated RPKM values of pri-miRNAs for the region from the start sites of the gene models to 500 nt downstream of the end of the gene models (Figure S6A and Table S4). These data showed that the tendency toward increased accumulation of the 3′ region of pri-miRNAs observed in *fry1-6* was also detected in *xrn3-3*, *xrn2xrn3*, and *xrn3xrn4* mutants (Figure 6B), suggesting that XRN3 also provides a critical activity for degrading 3′ RNA remnants of pri-miRNAs.

We performed qRT-PCR analysis of pri-miRNAs in all genotypes to further verify our hypothesis that XRN3 degrades 3′ RNA remnants of pri-miRNAs (Figure 6C). Two primer sets were used: a first primer set detected accumulation of the 5′ pri-miRNA region (pri-miRNA_5′), including the full stem loop; a second primer set detected accumulation of the 3′ region (pri-miRNA_3′), including the entire pri-miRNA and the 3′ remnants of DCL1-mediated cleavage (Table S1). The results revealed that, irrespective of accumulation of the pri-miRNA_5′ regions, accumulation of pri-miR160a_3′, pri-miR168a_3′, and pri-miR172a_3′ were clearly increased in *xrn3-3*, *xrn2xrn3*, and *fry1-6* and slightly increased in *xrn3xrn4* compared with those in wild-type. By contrast, it was difficult to judge whether accumulation of pri-miR156c_3′, pri-miR158a_3′, and pri-miR164b_3′ were dependent on XRN3 activity in the RT-PCR data, although increased accumulation of these transcripts was faintly detected in a few *xrn3-3* allele-containing plants in the RNA-Seq data (Figure S6B and Table S5). This result is likely due to the remaining XRN3 activity in *xrn3-3*, which is enough to repress the accumulation of the 3′ remnants of pri-miRNAs, preventing examination of whether other exoribonucleases act redundantly to repress the 3′ remnants. Taken together, the results indicate that XRN3 degrades unnecessary 3′ remnants of DCL1-mediated cleavage of pri-miRNAs.

### Lithium induces accumulation of 3′ extension transcripts

**Figure 7:**
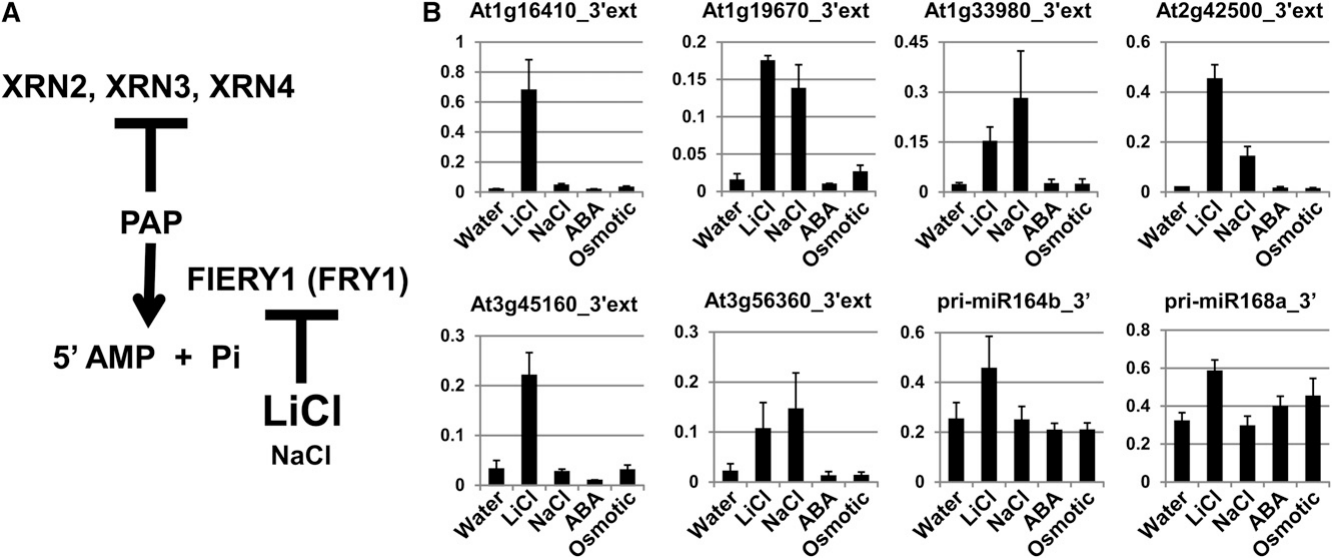
Lithium induces accumulation of 3′ extension transcripts. (A) Model for inhibition of FIERY1 activity by lithium. (B) Quantitative RT-PCR analysis of 3′ extensions and pri-miRNA_3′s under some stressful conditions. Wild-type seedlings were soaked in water, 200 mM LiCl, 200 mM NaCl, 100 μM abscisic acid (ABA), or 300 mM mannitol solutions, and incubated for 5 hr.

A previous report showed that lithium ion could inhibit activity of HAL2, a yeast FRY1 ortholog, and then deplete activity of exoribonucleases, such as Rat1 and Xrn1, which induced accumulation of many of non-coding RNAs in yeast (Dichtl *et al.* 1997; Van Dijk *et al.* 2011). It was also reported that lithium and sodium ions could inhibit the recombinant FRY1 protein from catalyzing PAP, although the effect of sodium was weaker than that of lithium (Xiong *et al.* 2004). The depletion of FRY1 activity by lithium in wild-type plants may induce accumulation of 3′ extension transcripts through suppression of XRN exoribonuclease activities including XRN3 (Figure 7A). To test this hypothesis, accumulation of some 3′ extensions and pri-miRNA_3′ under some abiotic stresses in wild-type seedlings was examined using quantitative RT-PCR. Accumulation of 3′ transcripts was strongly induced by LiCl stress in all cases and by NaCl stress in some cases (Figure 7B), regardless of accumulation of 5′ transcripts (Figure S7). This result supports the idea that depletion of FRY1 activity leads to accumulation of the 3′ transcripts by following suppression of XRN activities.

## Discussion

Using two genome-wide technologies, tiling arrays and RNA-Seq, we report the identification of transcript extensions from the 3′ ends of thousands of genes as well as 3′ remnants of pri-miRNAs that accumulate in *fry1-6* mutants. We also observed accumulation of these non-coding RNAs in *xrn3-3* mutants. These studies may suggest that XRN3 and FRY1 act cooperatively to eliminate two kinds of non-coding RNAs in plants: 3′ extensions from transcribed mRNAs and 3′ remnants of pri-miRNAs (Figure S8). This finding is supported by the fact that accumulation of the 3′ transcripts was induced in wild-type plant under high-salinity stresses that are inhibitors of FRY1 activity (Figure 7). Most notably, this is the first report of endogenous XRN3-specific activity in plants and genome-wide base resolution identification of thousands of 3′ gene transcript extensions.

### Gene transcript extensions are equivalent to 3′ read-through products

In yeast, the exoribonuclease Rat1 degrades 3′ read-through products that are transcribed by RNA polymerase II after endonucleolytic cleavage of the mRNA at the poly(A) site, and transcription terminates upon complete degradation by Rat1 (Kim *et al.* 2004; West *et al.* 2004). This Rat1-mediated reaction is closely coupled with transcription. It is very likely that the 3′ extensions detected in *fry1-6* mutants are equivalent to such 3′ read-through products and that only XRN3 (not XRN2 or XRN4) exclusively possesses this Rat1 function which can terminate the transcription reaction in *Arabidopsis*, regardless of the fact that XRN2 and XRN4 are also Rat1 orthologs (Kastenmayer and Green 2000). This indicates that some yeast Rat1 functions are divided into two proteins, XRN2 and XRN3, in *Arabidopsis*.

### XRN2 and XRN4 are not fully redundant with XRN3 for all functions

It was previously reported that XRN3 acts redundantly with XRN2 and XRN4 to suppress RNA silencing, to eliminate some loop structures of pri-miRNAs, and to process pre-ribosomal RNAs (Gy *et al.* 2007; Zakrzewska-Placzek *et al.* 2010). Interestingly, XRN2, another Rat1 ortholog that localizes to the nucleus together with XRN3, as well as cytoplasmic XRN4 compensate poorly for reduced XRN3 activity in the turnover of 3′ extensions, although it is likely that they redundantly act with XRN3 in a limited number of cases (Figure 4C, *eg* At3g45160). This observation supports the idea that elimination of unnecessary 3′ transcripts is the primary role of XRN3.

RNA-Seq data showed that the numbers of 3′ extension transcripts deregulated in *xrn3xrn4* (392) and *xrn2xrn3* (473) are lower than those deregulated in the *xrn3-3* single mutant (528). This indicates that the three *xrn* mutations genetically interfere with each other’s function(s) in an unknown manner. Interestingly, previous studies have shown that whereas the *xrn2xrn3* double mutant is infertile (Gy *et al.* 2007), the *xrn2xrn3xrn4* triple mutant is, in fact, fertile (Hirsch *et al.* 2011). These findings may be partly consistent with our results from qRT-PCR experiments, which reveal that *xrn2* and *xrn4* mutations can, in some cases, enhance or suppress the effect of the *xrn3-3* mutation on the accumulation of 3′ extension transcripts.

On the other hand, it is likely that XRN2, XRN4, or other exoribonucleases redundantly act with XRN3 to eliminate 3′ remnants of pri-miRNAs, because we could not detect significantly increased accumulation of pri-miR156c_3′, pri-miR158a, or pri-miR164b in qRT-PCR analysis. Furthermore, RNA-Seq data showed that some pri-miRNAs accumulated in the *xrn2xrn4* double mutant compared with wild-type. This observation provides support for the idea that, like *S. cerevisiae* Rat1, XRN3-mediated degradation of 3′ extensions is probably coupled with transcription and that degradation of 3′ remnants of pri-miRNAs is not necessarily coupled with transcription. If DCL1-mediated cleavage of pri-miRNA occurs before transcription terminates, XRN3 may capture the 5′ end of the 3′ remnant, whereas if DCL1-mediated cleavage occurs after transcription terminates, other exoribonucleases may participate in this degradation.

### Characteristics of the mRNAs with 3′extensions

Many, but not all, mRNAs showed 3′ extensions in the *xrn* or *fry1* mutants. When we examined whether mRNAs tailed with 3′ extensions possess specific characteristics, we noticed a significant number of the mRNAs with 3′ extensions had relatively higher RPKM values compared with all expressed genes. These data revealed that actively transcribed genes tend to possess 3′ extensions.

Importantly, mRNAs with 3′ extensions showed comparable expression in wild-type and *fry1-6*, regardless of increased accumulation of 3′ extensions. This result indicates that 3′ extensions themselves do not affect the expression levels of the 5′ mRNAs.

It has been proposed that elevated accumulation of PAP inhibits XRN3 activity, as well as XRN2 and XRN4 activities, in *fry1* and that 3′ extensions being transcribed could partially escape from degradation by reduced XRN3 activity in both *fry1* and *xrn3*. However, transcription might terminate at unknown downstream positions, such as poly(A) addition sites. *Arabidopsis* uses not only the AAUAAA consensus sequence conserved in all eukaryotes but also several other AU-rich consensus sequences as poly(A) signals that often appear in intergenic regions (Loke *et al.* 2005). This mechanism of alternative transcription termination could define the length of 3′ extensions in *fry1* and *xrn3* mutant backgrounds. Further studies are needed to identify why some mRNAs may possess 3′ extensions whereas others do not in these mutants.

### The role of XRN3- and FRY1-mediated non-coding RNA suppression

The RNA-binding proteins FCA and FPA are known as positive regulators of the floral transition by downregulating expression of the MADS box floral repressor FLC (Koornneef *et al.* 1991). A recent study using tiling analysis detected 3′ read-through signals at the 3′ ends of several genes in an *fcafpa* double mutant (Sonmez *et al.* 2011). However, these findings differ from our study in that the 3′ extensions we identified are generally independent molecules from 5′ mRNAs, whereas 3′ read-through products in *fcafpa* mutants are likely connected directly with mRNAs.

Sonmez *et al.* (2011) also found accumulation of transcripts of one transposon (At1TE93275) that was associated with reduced DNA methylation in *fcafpa*. Our study also revealed much greater accumulation of this antisense transcript originating from this transposon in *fry1-6*, *xrn2-4*, and *xrn4-6* compared with those in other backgrounds, as well as strongly reduced DNA methylation in *fry1-6*, indicating that regulation of this transposon is quite different from that of 3′ extensions, which is primarily mediated by XRN3 activity. In future studies, it will be interesting to reveal the relationship between FRY1- and XRN3-mediated transcription termination and the RNA-binding proteins FCA and FPA.

## Supplementary Material

Supporting Information
