# Supplementary material for: Surveillance of 3′ Noncoding Transcripts Requires FIERY1 and XRN3 in Arabidopsis
Source: G3 (Bethesda). 2012 Apr 1;2(4):487–98. doi: 10.1534/g3.111.001362 (PMC3337477; doi:10.1534/g3.111.001362)
Supplement: Supporting Information [file supp_2_4_487__index.html]

Supporting Information 

# Surveillance of 3′ Noncoding Transcripts Requires FIERY1 and XRN3 in *Arabidopsis*

## Supporting Information for Kurihara *et al*, 2012

**Files in this Data Supplement:**

- Supporting Information - Figures S1-S8 and Tables S1-S5 (PDF, 1.3 MB
- Figure S1 - Identification and characterization of 3' extensions in the *fry-1-6* mutant (PDF, 476 KB)
- Figure S2 - Characterization of mRNAs with 3' extensions (PDF, 235 KB)
- Figure S3 - Increased accumulation of 3' extensions in *xrn3-3* genotypes(PDF, 184 KB)
- Figure S4 - Expression of the antisense transcript of At1TE93275 in tiling array data and RNA-Seq data (PDF, 103 KB)
- Figure S5 - Effect of FRY1 and exoribonucleases on miRNA targets (PDF, 255 KB)
- Figure S6 - Effect of FRY1 and exoribonucleases on pri-miRNAs (PDF, 114 KB)
- Figure S7 - Quantitative RT-PCR analysis of 5' mRNAs and pri-miRNA\_5's of representitive genes after various plant stress treatments (PDF, 99 KB)
- Figure S8 - A proposed model for FRY1 and XRN3 (PDF, 88 KB)
- Table S1 - (.xls, 30 KB)
- Table S2 - (.xls, 495 KB)
- Table S3 - (.xls, 301 KB)
- Table S4 - (.xls, 26 KB)
- Table S5 - (.xls, 62 KB)
