# Supplementary material for: Surveillance of 3′ Noncoding Transcripts Requires FIERY1 and XRN3 in Arabidopsis
Source: G3 (Bethesda). 2012 Apr 1;2(4):487–98. doi: 10.1534/g3.111.001362 (PMC3337477; doi:10.1534/g3.111.001362)
Supplement: Supporting Information [file supp_2.4.487_001362SI.pdf]

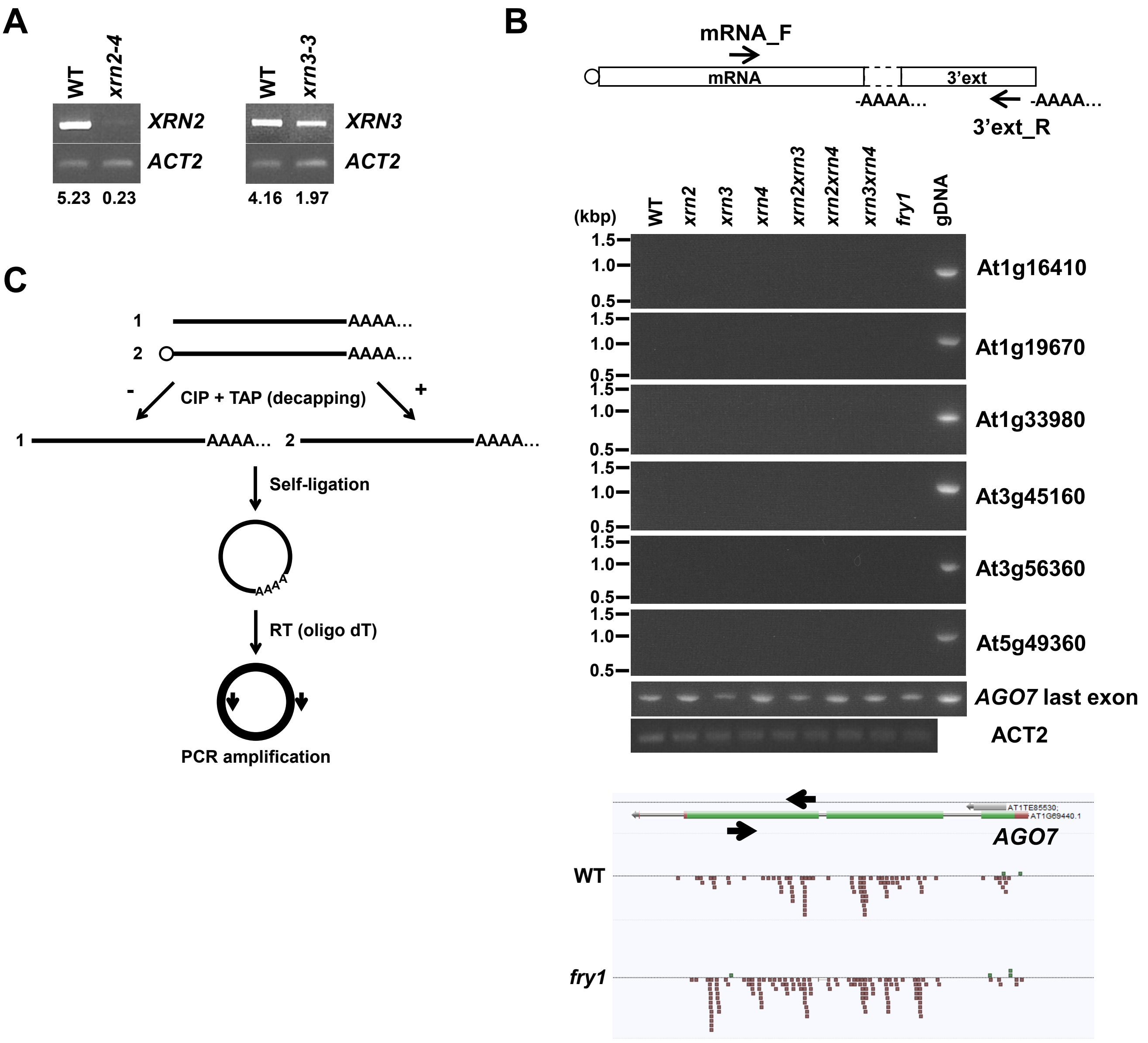

Figure S1 Identification and characterization of 3' extensions in the *fry1-6* mutant. (A) Quantitative RT-PCR analysis of *XRN2* and *XRN3* expressions in *xrn2-4* and *xrn3-3*, respectively. *ACT2* mRNA was used as an internal control. The relative accumulation normalized to *ACT2* expression is shown under the panels. (B) Quantitative RT-PCR analysis was used to determine whether 5' mRNA and 3' extensions are one transcript or two separate transcripts. Positions of primers used here are shown in the illustration of the mRNA and the 3' extension. PCR was carried out using Phire Hot Start DNA polymerase for 32 cycles. The fragment (996 nt) of the 3rd exon of *AGO7* mRNA was used as a positive control, because its expression is relatively-lower and the 3rd exon is more than 1,000nt in length as shown in the lower panel (arrows are primers). (C) A schematic of the experimental procedure of the self-ligation-mediated RT-PCR analysis. The results were shown in Figure 2B.
CIP, Calf Intestinal Phosphatase ;TAP, Tobacco Acid Pyrophosphatase.

A

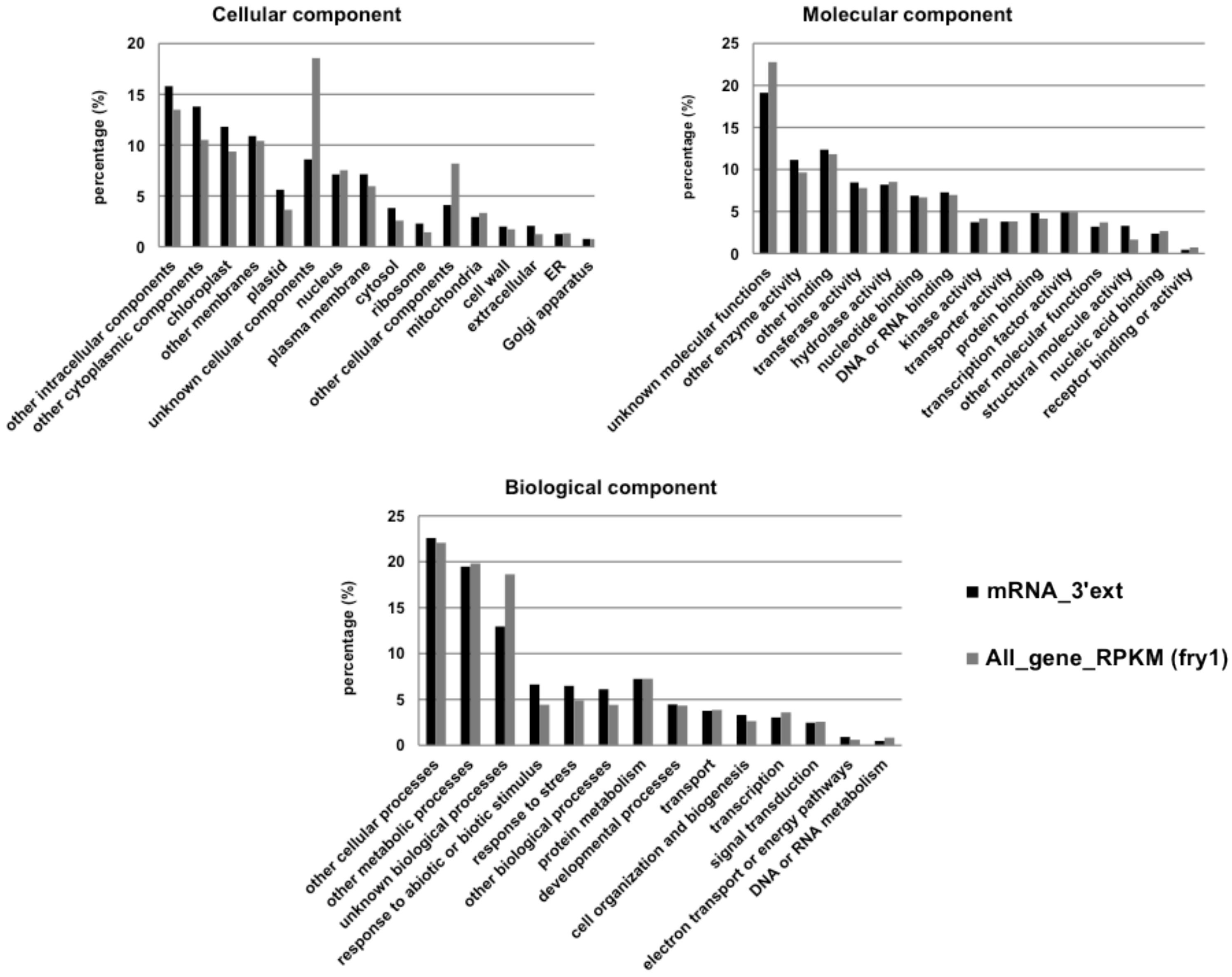

B

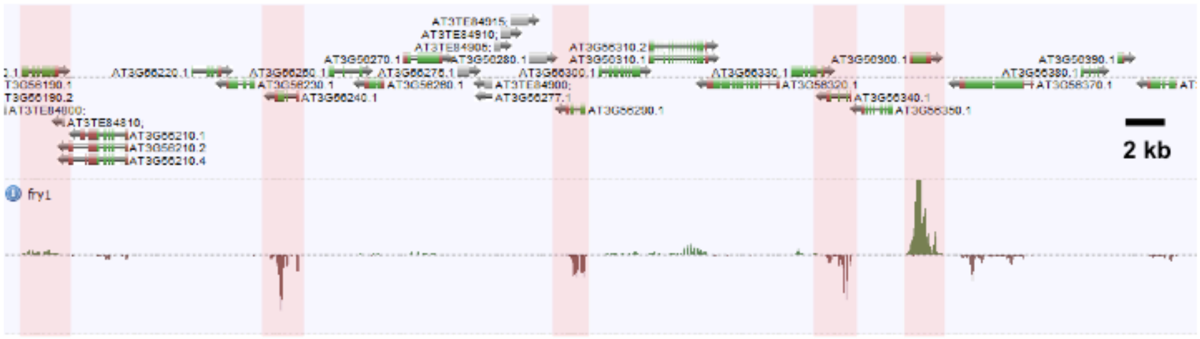

Figure S2 Characterization of mRNAs with 3′ extensions. (A) Classification of mRNAs tailed with 2,230 3′ extensions based on their gene ontology (GO) annotations. Population comprising all expressed genes with RPKM values in *fry1-6* was used as a control. (B) Loci on which there are 5 mRNAs tailed with 3′ extensions. Their expression levels are relatively higher than those of neighboring mRNAs.

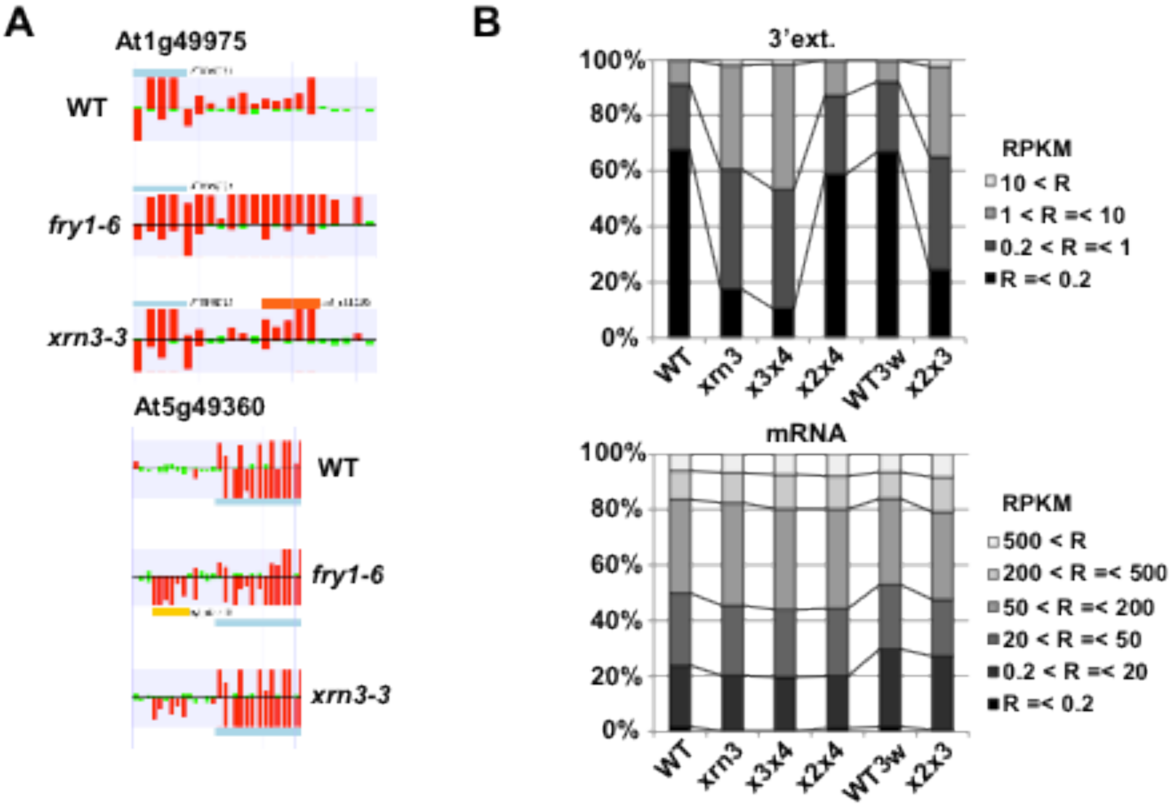

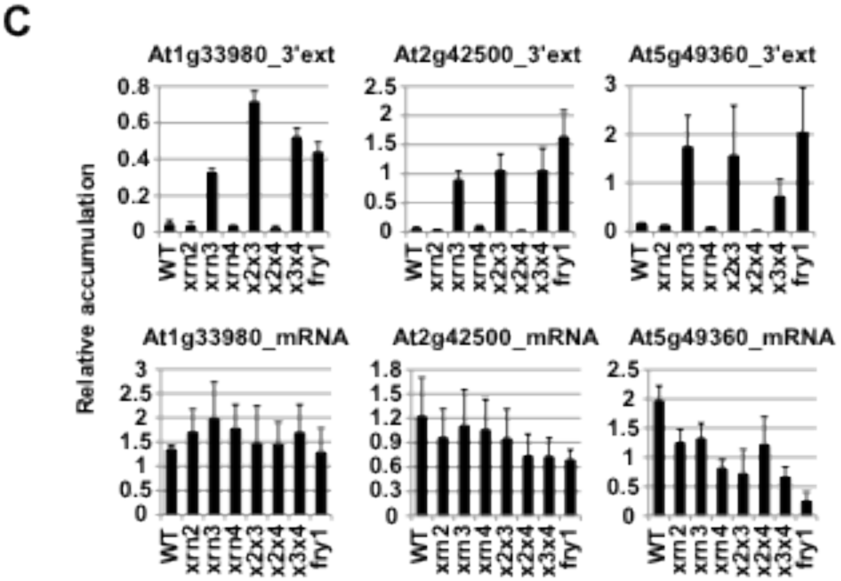

Figure S3 Increased accumulation of 3' extensions in *xrn3-3* genotypes. (A) Examples of 3' extensions identified by the ARTADE program (Toyoda and Shinozaki 2005) in tiling array data from *xrn3-3*. Vertical red bars indicate signal intensities on each probe. Horizontal blue regions are exons in the gene model. Horizontal orange regions are predicted 3' extensions. (B) Comparison of accumulation profiles of 2,230 3' extensions identified in *fry1-6* and the 5' mRNAs among wild type, *xrn3-3*, *xrn3xrn4*, *xrn2xrn4*, 3-week-old wild type and *xrn2xrn3*. RPKM values of 3' extensions and the mRNAs were classified into four and six categories respectively. (C) Additional quantitative RT-PCR analysis of three 3' extensions and the 5' mRNAs. Vertical axes show relative accumulation normalized against *ACT2* expression. x2x3 = xrn2xrn3; x2x4 = xrn2xrn4; x3x4 = xrn3xrn4. (D) Examples of 3' extensions at the 3' end of At1g19670 and At3g45160. Pink-marked regions are 3' extensions. The length of black scale bar is 500nt.

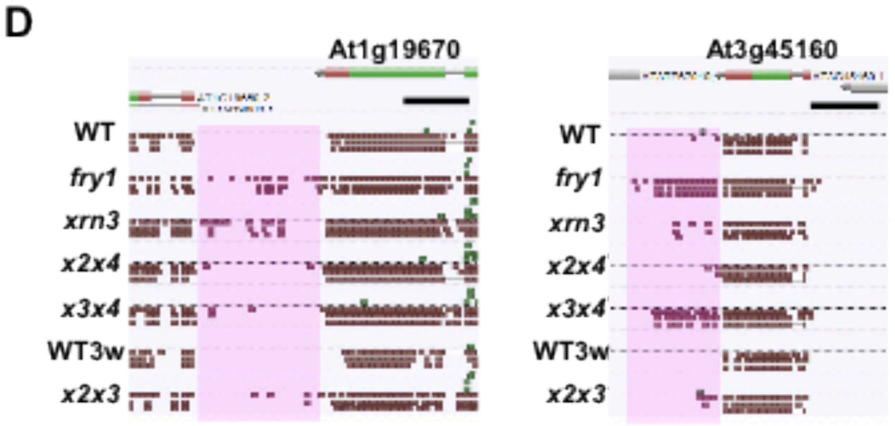

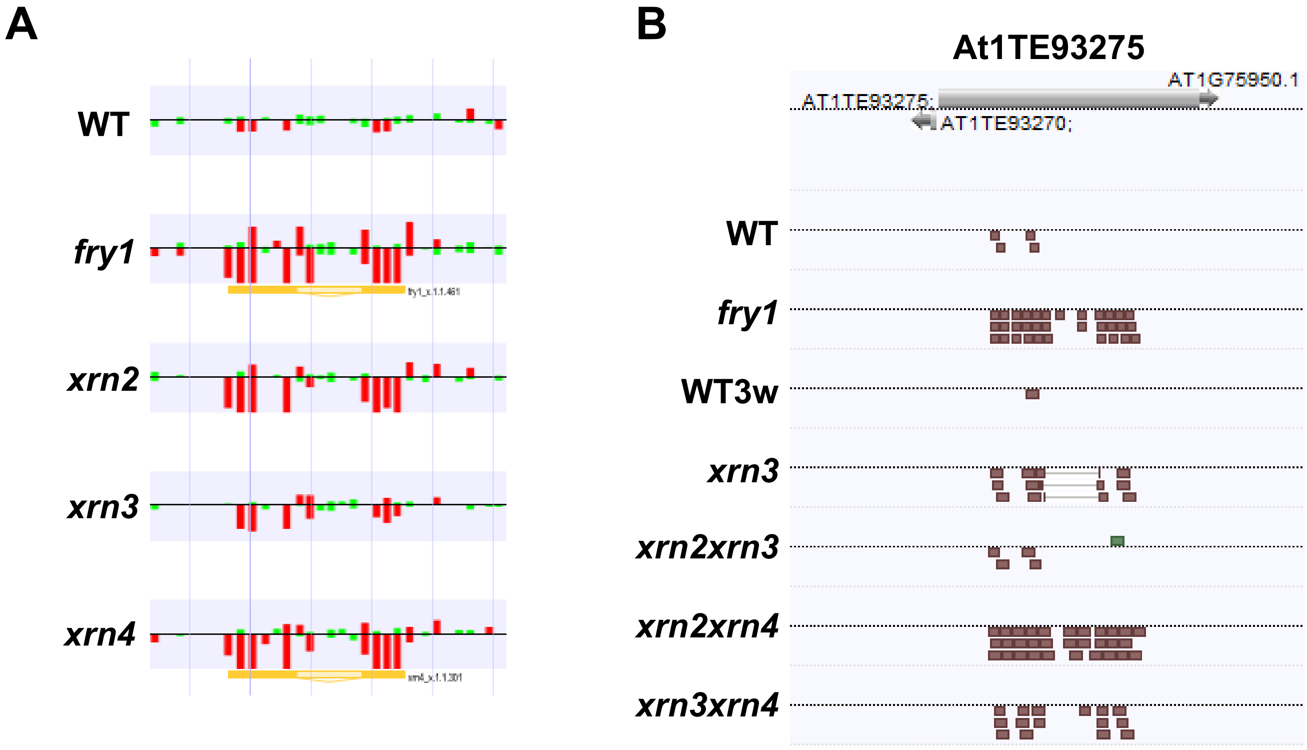

Figure S4 Expression of the antisense transcript of At1TE93275 in tiling array data (A) and RNA-Seq data (B).

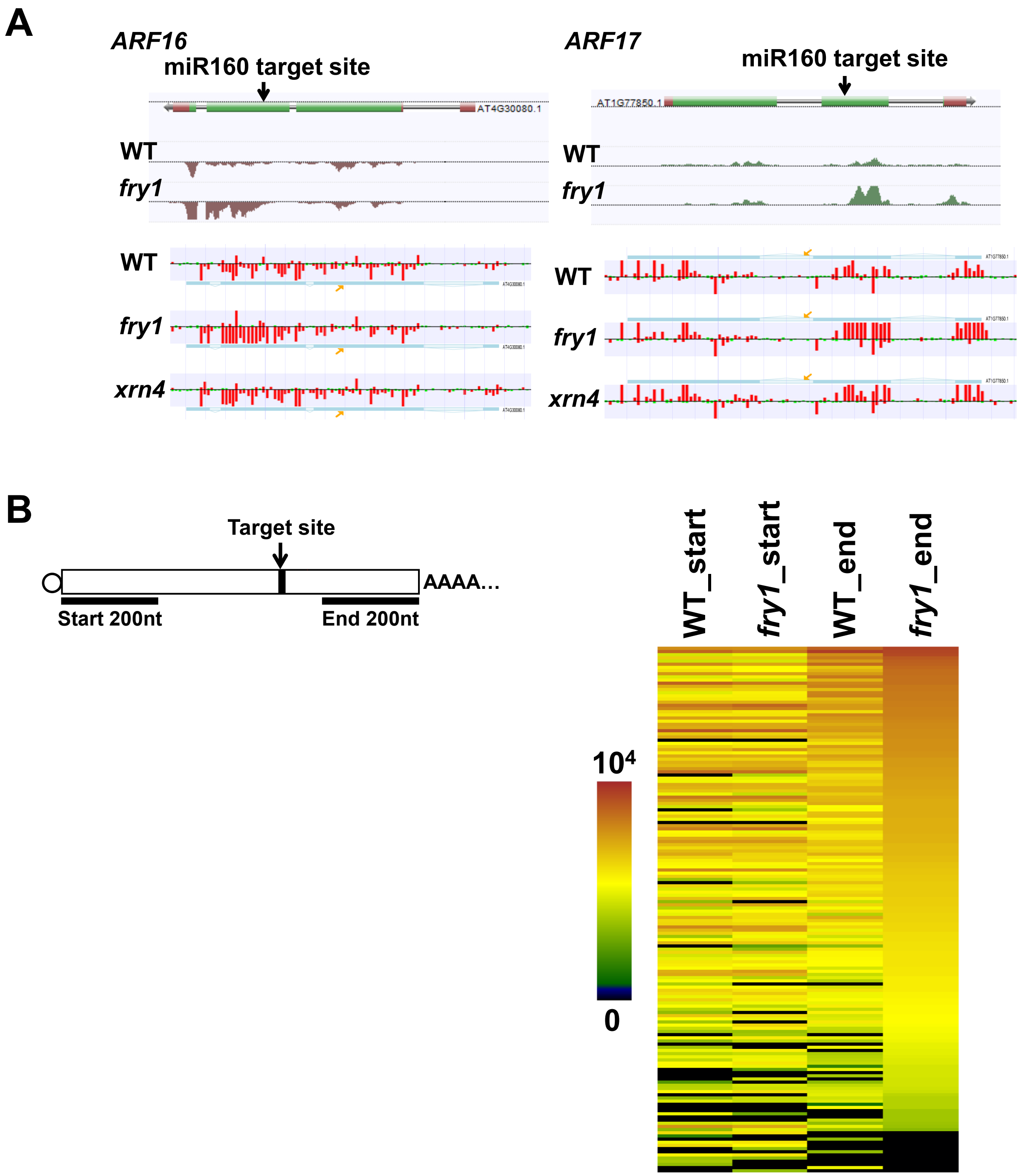

Figure S5 Effect of FRY1 and exoribonucleases on miRNA targets. (A) Examples of miRNA targets ARF16 and ARF17. Both mRNAs are targeted by miR160. RNA-Seq (upper) and tiling array data (lower) are shown. Arrows indicate the position of the miR160 target sites. Accumulation of 3’ products of miRNA-mediated cleavage of the targets were increased in *fry1-6* and *xrn4-6*. (B) A Heat map of 165 miRNA-targeted mRNAs with RPKM values. RPKM values were calculated in two ranges; 200nt downstream of start sites of the genes and 200nt upstream of 3’ ends of the genes. The transcripts are sorted according to RPKM values of 3’ ends in *fry1-6*.

A

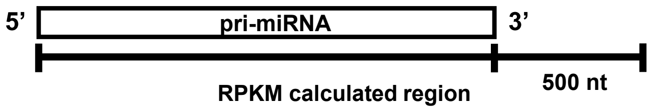

B

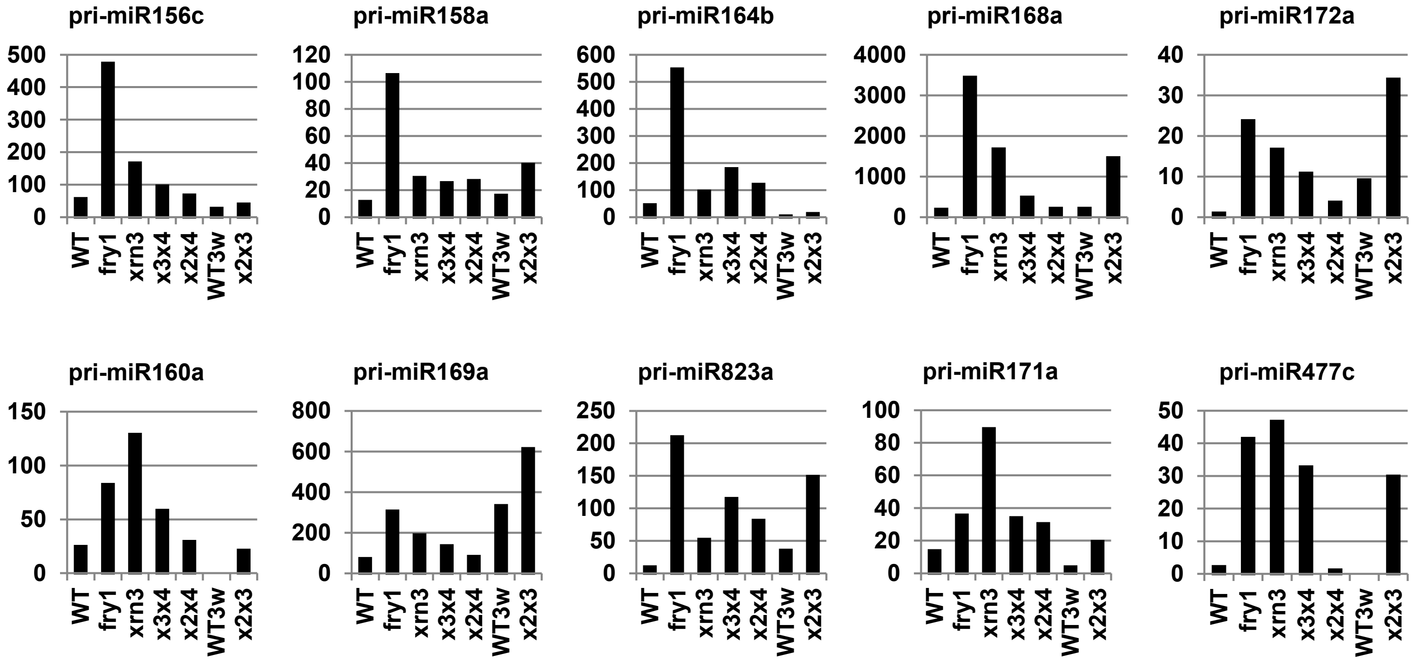

Figure S6 Effect of FRY1 and exoribonucleases on pri-miRNAs. (A) A schematic of the region used for calculation of RPKM values for pri-miRNAs. (B) RNA-Seq data of ten pri-miRNAs. Averages of RPKM values of 3 replicates of RNA-Seq in wild type and *fry1-6* and 2 replicates of RNA-Seq in *xrn3-3*, *xrn3xrn4*, 3-week-old wild type and *xrn2xrn3* were shown.

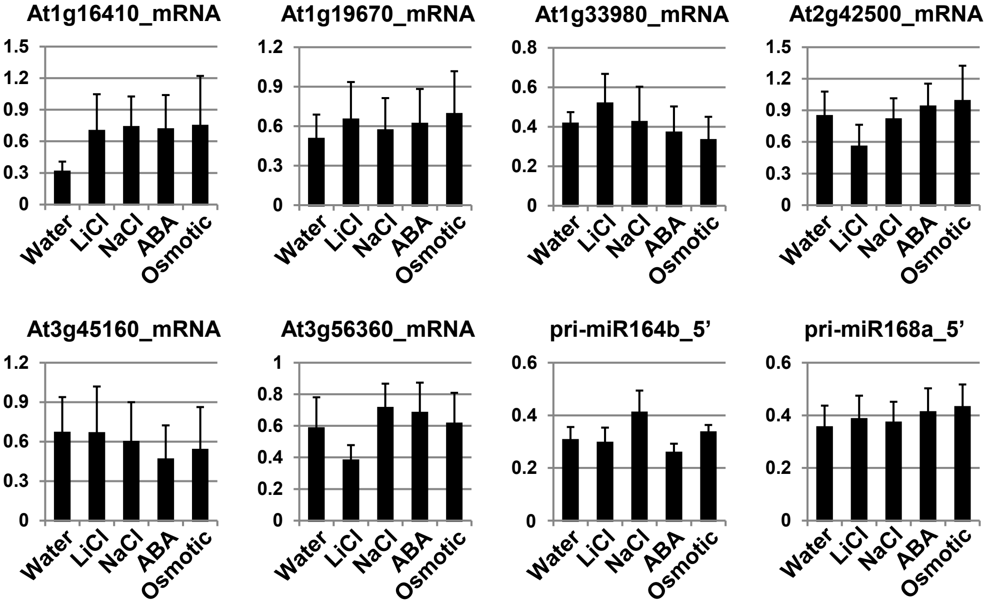

Figure S7 Quantitative RT-PCR analysis of 5’ mRNAs and pri-miRNA_5’s of representitive genes after various plant stress treatments. Vertical axes show relative accumulation normalized against *ACT2* expression.

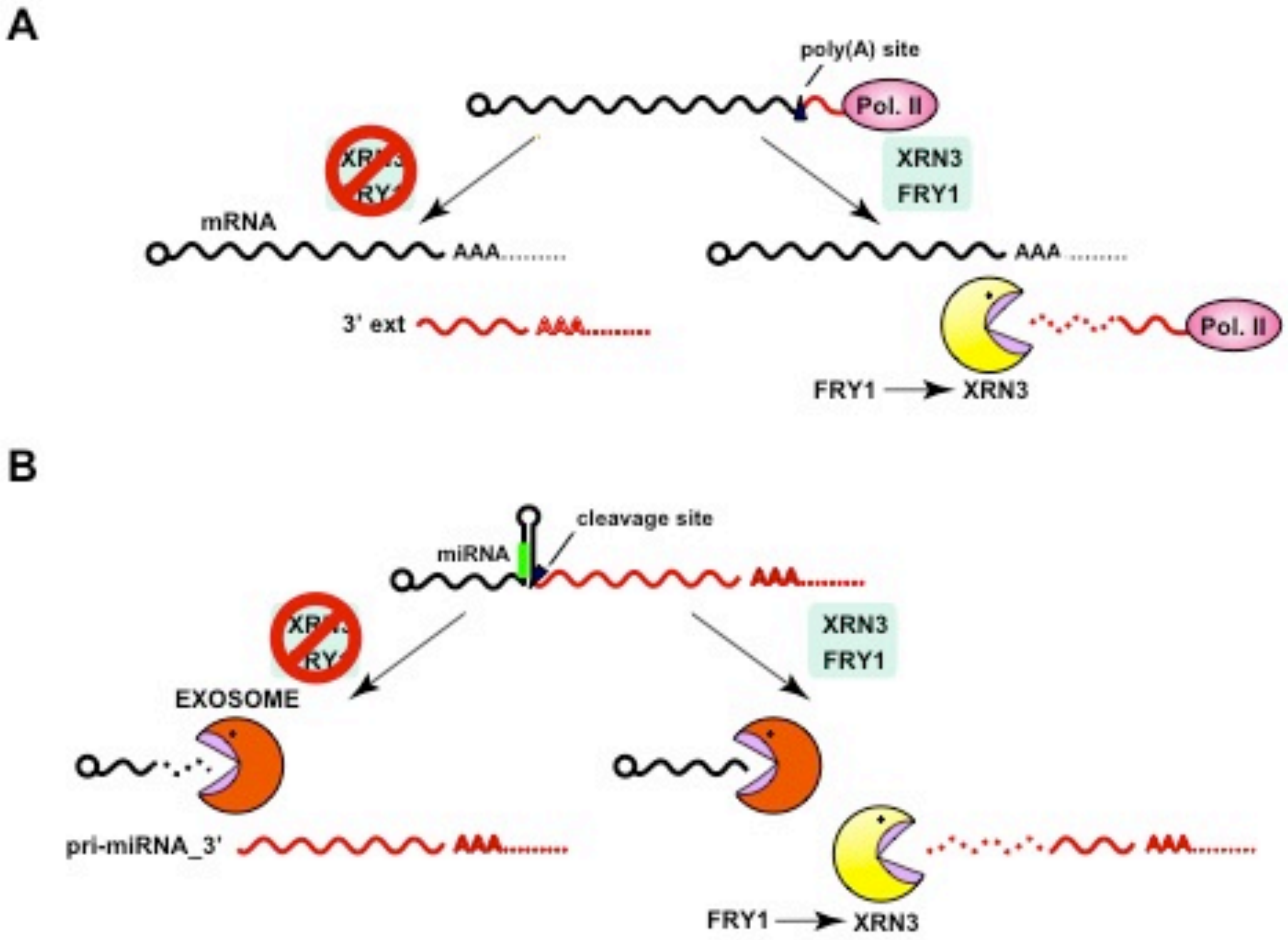

Figure S8 A proposed model for FRY1 and XRN3. (A) The 3' extension after cleavage at poly(A) sites during transcription is degraded in a 5'-to-3' direction by XRN3. (B) The 3' remnant of DCL1-mediated cleavage of pri-miRNA is degraded in a 5'-to-3' direction by XRN3.

Supporting Tables

Tables S1-S5 are available for download at http://www.g3journal.org/lookup/suppl/doi:10.1534/g3.111.001362/-/DC1 as Excel files.
