# Supplementary material for: Surveillance of 3′ Noncoding Transcripts Requires FIERY1 and XRN3 in Arabidopsis
Source: G3 (Bethesda). 2012 Apr 1;2(4):487–98. doi: 10.1534/g3.111.001362 (PMC3337477; doi:10.1534/g3.111.001362)
Supplement: Supporting Information [file supp_2.4.487_FigureS1.pdf]

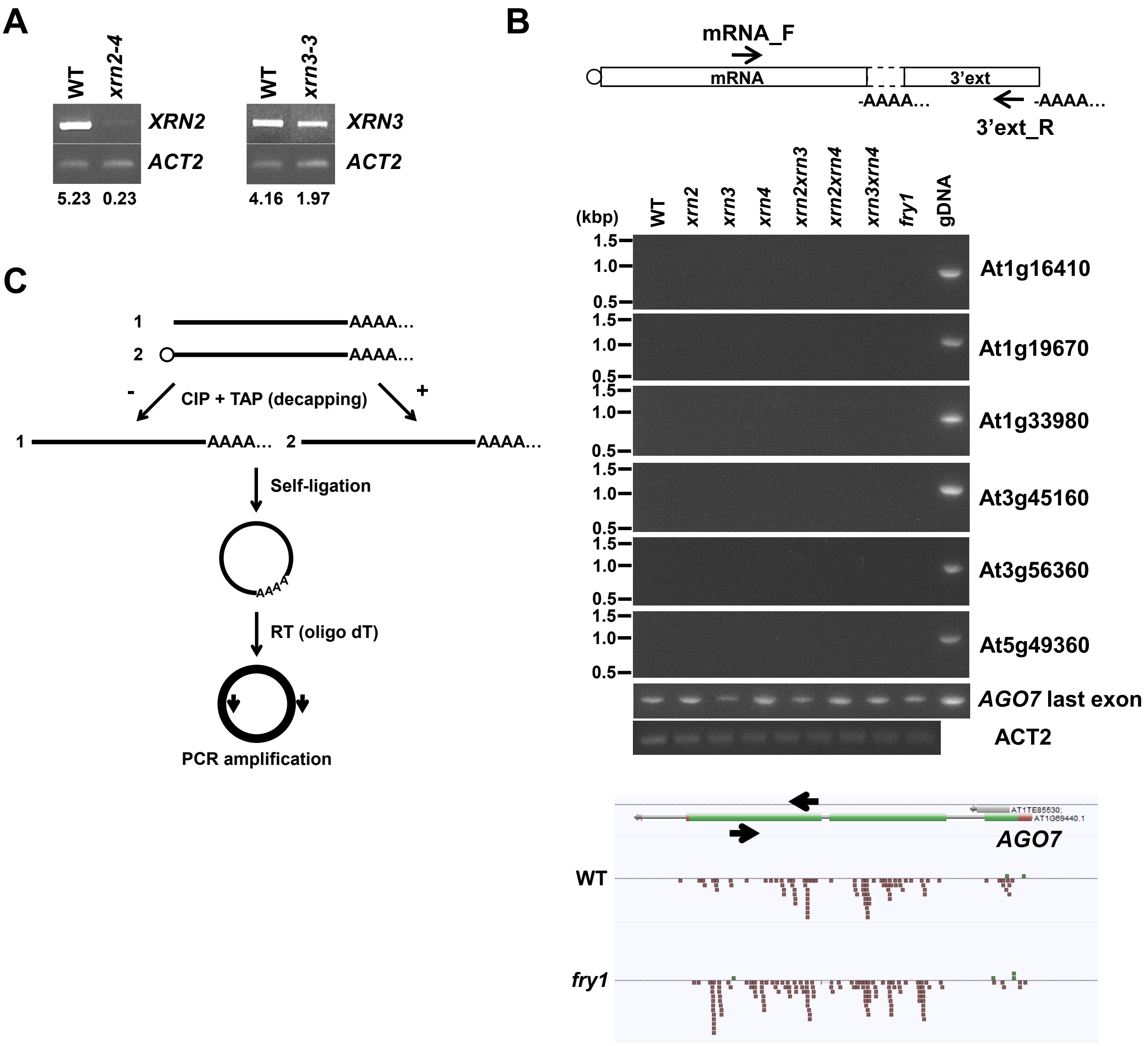

Figure S1 Identification and characterization of 3' extensions in the *fry1-6* mutant. (A) Quantitative RT-PCR analysis of *XRN2* and *XRN3* expressions in *xrn2-4* and *xrn3-3*, respectively. *ACT2* mRNA was used as an internal control. The relative accumulation normalized to *ACT2* expression is shown under the panels. (B) Quantitative RT-PCR analysis was used to determine whether 5' mRNA and 3' extensions are one transcript or two separate transcripts. Positions of primers used here are shown in the illustration of the mRNA and the 3' extension. PCR was carried out using Phire Hot Start DNA polymerase for 32 cycles. The fragment (996 nt) of the 3rd exon of *AGO7* mRNA was used as a positive control, because its expression is relatively-lower and the 3rd exon is more than 1,000nt in length as shown in the lower panel (arrows are primers). (C) A schematic of the experimental procedure of the self-ligation-mediated RT-PCR analysis. The results were shown in Figure 2B.
CIP, Calf Intestinal Phosphatase ;TAP, Tobacco Acid Pyrophosphatase.
