# Supplementary material for: Surveillance of 3′ Noncoding Transcripts Requires FIERY1 and XRN3 in Arabidopsis
Source: G3 (Bethesda). 2012 Apr 1;2(4):487–98. doi: 10.1534/g3.111.001362 (PMC3337477; doi:10.1534/g3.111.001362)
Supplement: Supporting Information [file supp_2.4.487_FigureS2.pdf]

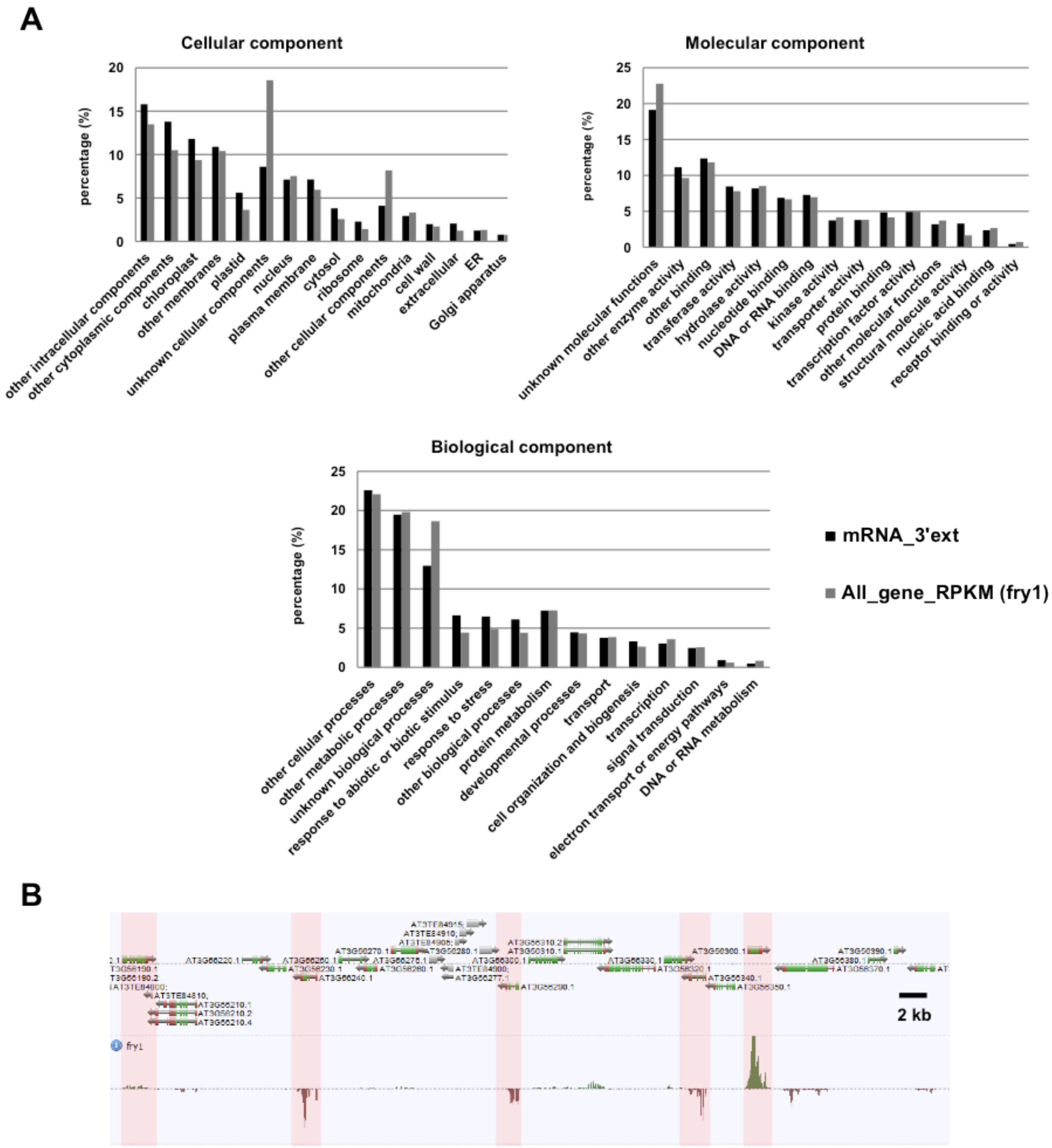

Figure S2 Characterization of mRNAs with 3′ extensions. (A) Classification of mRNAs tailed with 2,230 3′ extensions based on their gene ontology (GO) annotations. Population comprising all expressed genes with RPKM values in *fry1-6* was used as a control. (B) Loci on which there are 5 mRNAs tailed with 3′ extensions. Their expression levels are relatively higher than those of neighboring mRNAs.
