# Supplementary material for: Surveillance of 3′ Noncoding Transcripts Requires FIERY1 and XRN3 in Arabidopsis
Source: G3 (Bethesda). 2012 Apr 1;2(4):487–98. doi: 10.1534/g3.111.001362 (PMC3337477; doi:10.1534/g3.111.001362)

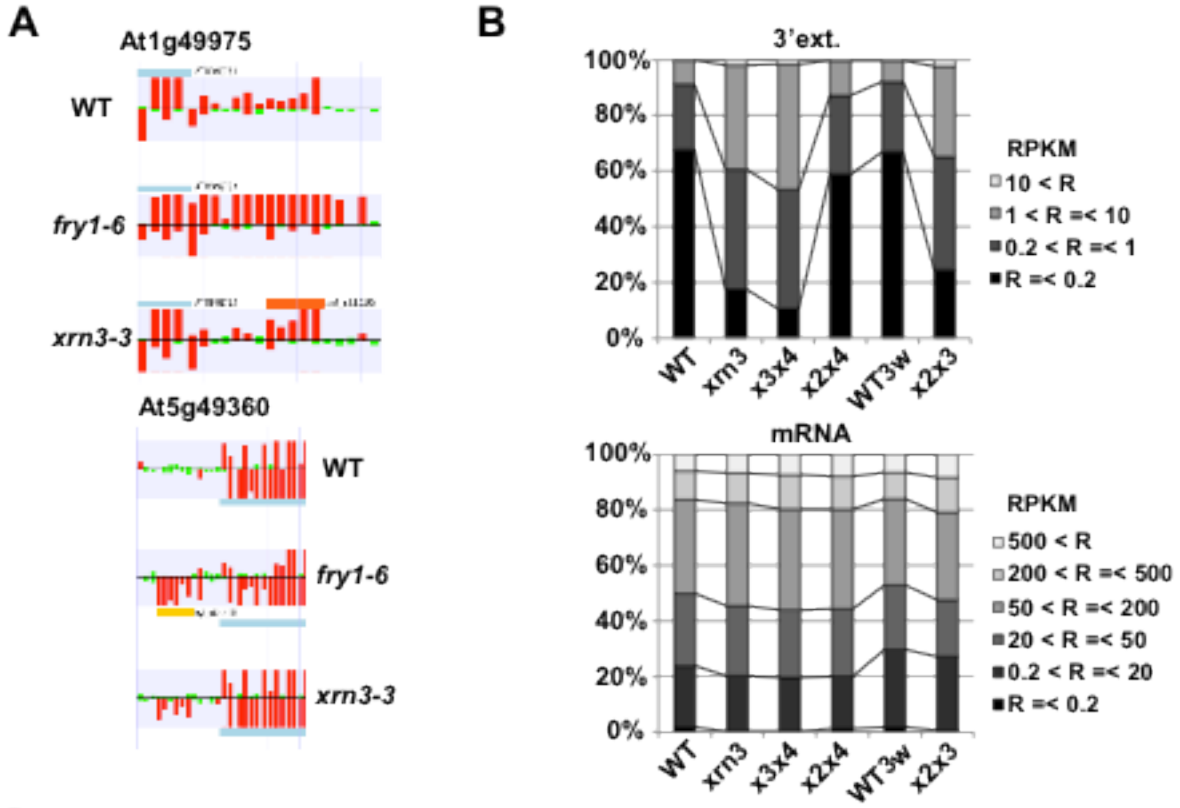

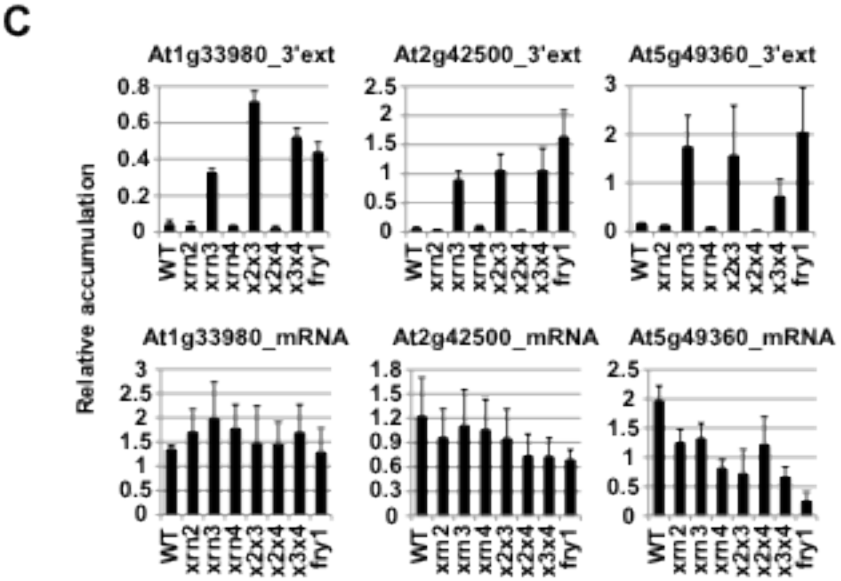

Figure S3 Increased accumulation of 3' extensions in *xrn3-3* genotypes. (A) Examples of 3' extensions identified by the ARTADE program (Toyoda and Shinozaki 2005) in tiling array data from *xrn3-3*. Vertical red bars indicate signal intensities on each probe. Horizontal blue regions are exons in the gene model. Horizontal orange regions are predicted 3' extensions. (B) Comparison of accumulation profiles of 2,230 3' extensions identified in *fry1-6* and the 5' mRNAs among wild type, *xrn3-3*, *xrn3xrn4*, *xrn2xrn4*, 3-week-old wild type and *xrn2xrn3*. RPKM values of 3' extensions and the mRNAs were classified into four and six categories respectively. (C) Additional quantitative RT-PCR analysis of three 3' extensions and the 5' mRNAs. Vertical axes show relative accumulation normalized against *ACT2* expression. x2x3 = xrn2xrn3; x2x4 = xrn2xrn4; x3x4 = xrn3xrn4. (D) Examples of 3' extensions at the 3' end of At1g19670 and At3g45160. Pink-marked regions are 3' extensions. The length of black scale bar is 500nt.

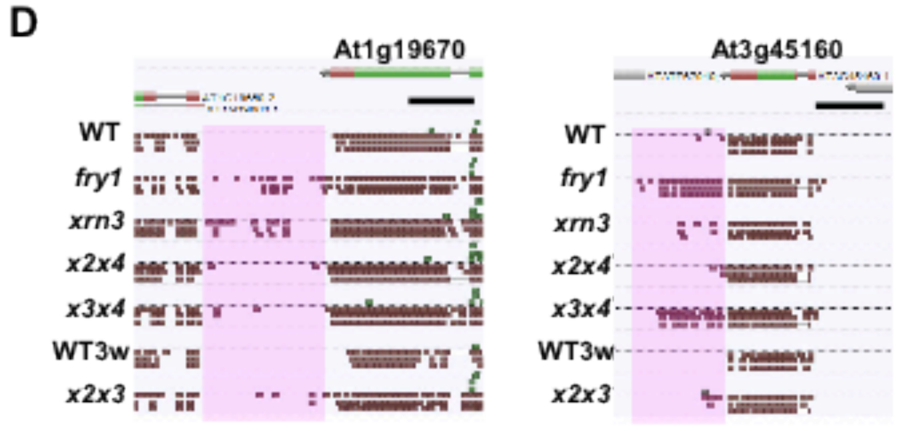

Supplement: Supporting Information [file supp_2.4.487_FigureS3.pdf]
