# Supplementary material for: Surveillance of 3′ Noncoding Transcripts Requires FIERY1 and XRN3 in Arabidopsis
Source: G3 (Bethesda). 2012 Apr 1;2(4):487–98. doi: 10.1534/g3.111.001362 (PMC3337477; doi:10.1534/g3.111.001362)
Supplement: Supporting Information [file supp_2.4.487_FigureS4.pdf]

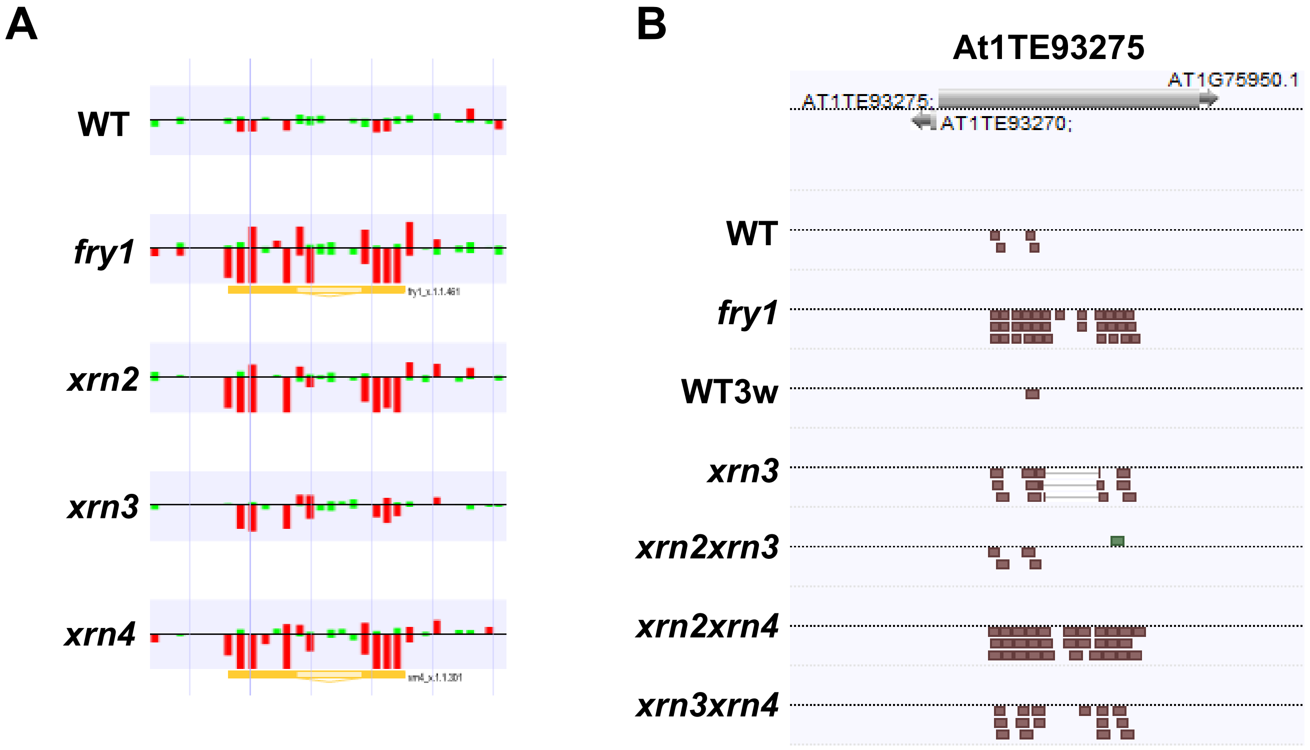

Figure S4 Expression of the antisense transcript of At1TE93275 in tiling array data (A) and RNA-Seq data (B).
