# Supplementary material for: Surveillance of 3′ Noncoding Transcripts Requires FIERY1 and XRN3 in Arabidopsis
Source: G3 (Bethesda). 2012 Apr 1;2(4):487–98. doi: 10.1534/g3.111.001362 (PMC3337477; doi:10.1534/g3.111.001362)
Supplement: Supporting Information [file supp_2.4.487_FigureS5.pdf]

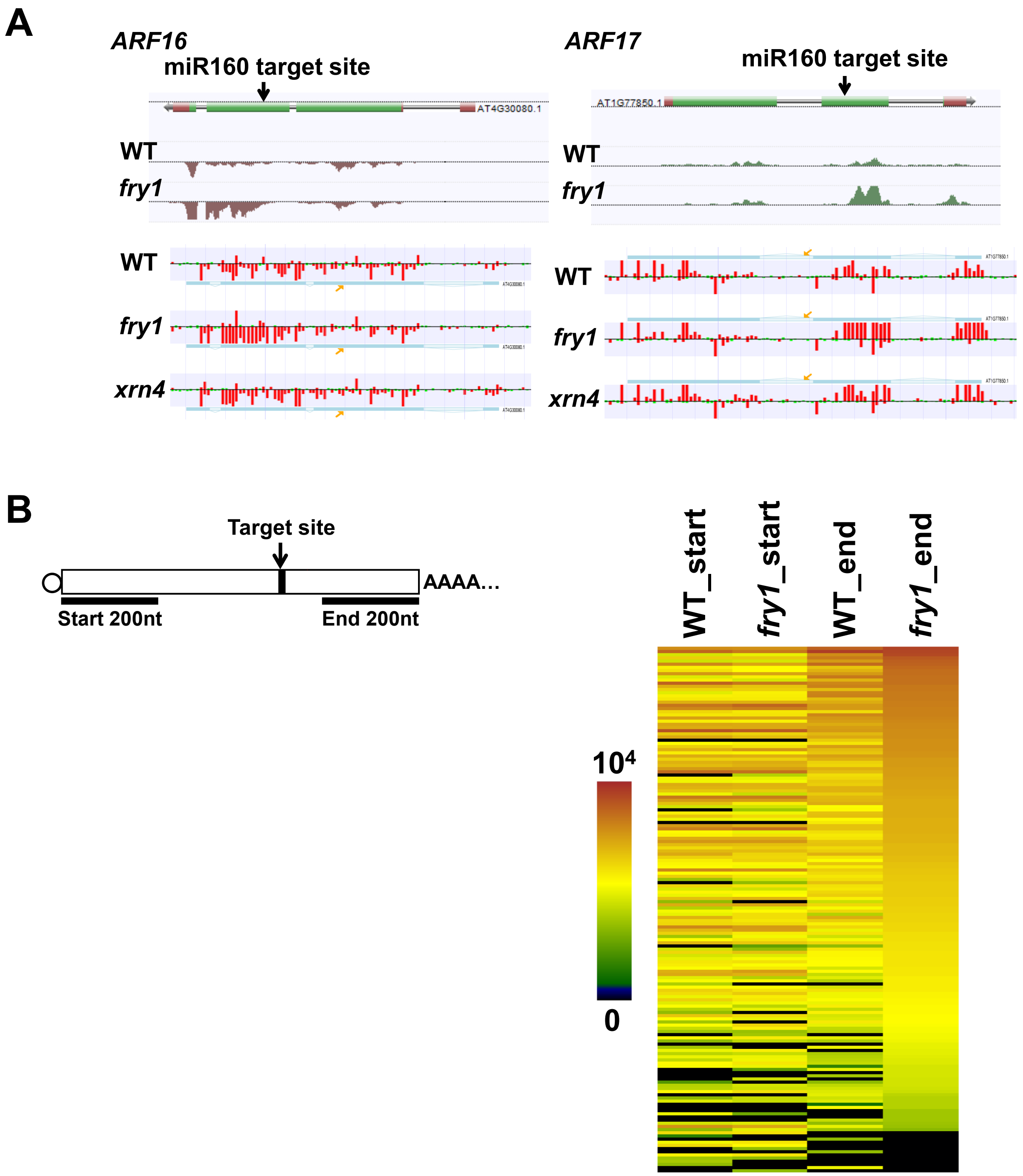

Figure S5 Effect of FRY1 and exoribonucleases on miRNA targets. (A) Examples of miRNA targets ARF16 and ARF17. Both mRNAs are targeted by miR160. RNA-Seq (upper) and tiling array data (lower) are shown. Arrows indicate the position of the miR160 target sites. Accumulation of 3’ products of miRNA-mediated cleavage of the targets were increased in *fry1-6* and *xrn4-6*. (B) A Heat map of 165 miRNA-targeted mRNAs with RPKM values. RPKM values were calculated in two ranges; 200nt downstream of start sites of the genes and 200nt upstream of 3’ ends of the genes. The transcripts are sorted according to RPKM values of 3’ ends in *fry1-6*.
