# Supplementary material for: Surveillance of 3′ Noncoding Transcripts Requires FIERY1 and XRN3 in Arabidopsis
Source: G3 (Bethesda). 2012 Apr 1;2(4):487–98. doi: 10.1534/g3.111.001362 (PMC3337477; doi:10.1534/g3.111.001362)
Supplement: Supporting Information [file supp_2.4.487_FigureS6.pdf]

A

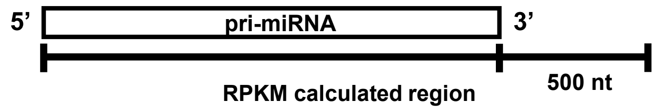

B

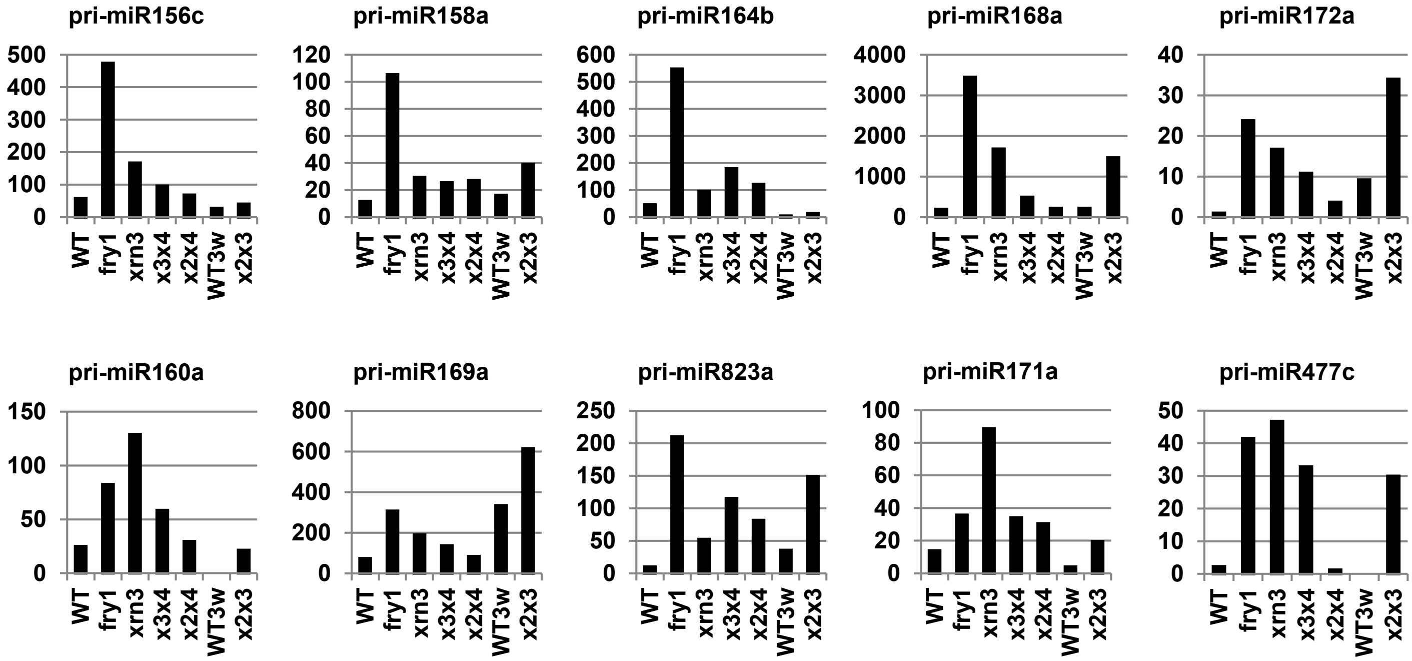

Figure S6 Effect of FRY1 and exoribonucleases on pri-miRNAs. (A) A schematic of the region used for calculation of RPKM values for pri-miRNAs. (B) RNA-Seq data of ten pri-miRNAs. Averages of RPKM values of 3 replicates of RNA-Seq in wild type and *fry1-6* and 2 replicates of RNA-Seq in *xrn3-3*, *xrn3xrn4*, 3-week-old wild type and *xrn2xrn3* were shown.
