# Supplementary material for: Surveillance of 3′ Noncoding Transcripts Requires FIERY1 and XRN3 in Arabidopsis
Source: G3 (Bethesda). 2012 Apr 1;2(4):487–98. doi: 10.1534/g3.111.001362 (PMC3337477; doi:10.1534/g3.111.001362)
Supplement: Supporting Information [file supp_2.4.487_FigureS7.pdf]

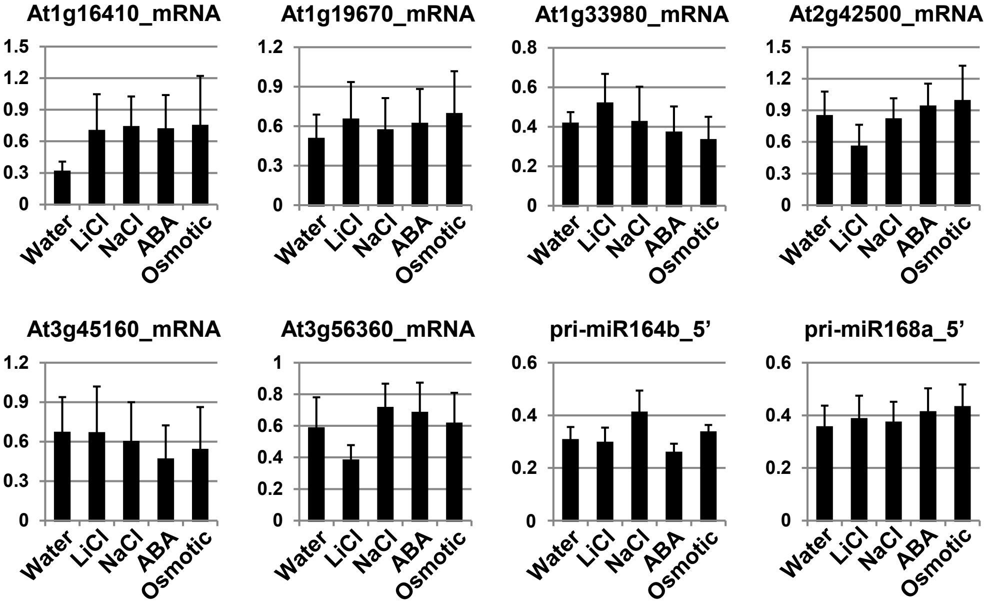

Figure S7 Quantitative RT-PCR analysis of 5’ mRNAs and pri-miRNA_5’s of representitive genes after various plant stress treatments. Vertical axes show relative accumulation normalized against *ACT2* expression.
