# Supplementary material for: Surveillance of 3′ Noncoding Transcripts Requires FIERY1 and XRN3 in Arabidopsis
Source: G3 (Bethesda). 2012 Apr 1;2(4):487–98. doi: 10.1534/g3.111.001362 (PMC3337477; doi:10.1534/g3.111.001362)
Supplement: Supporting Information [file supp_2.4.487_FigureS8.pdf]

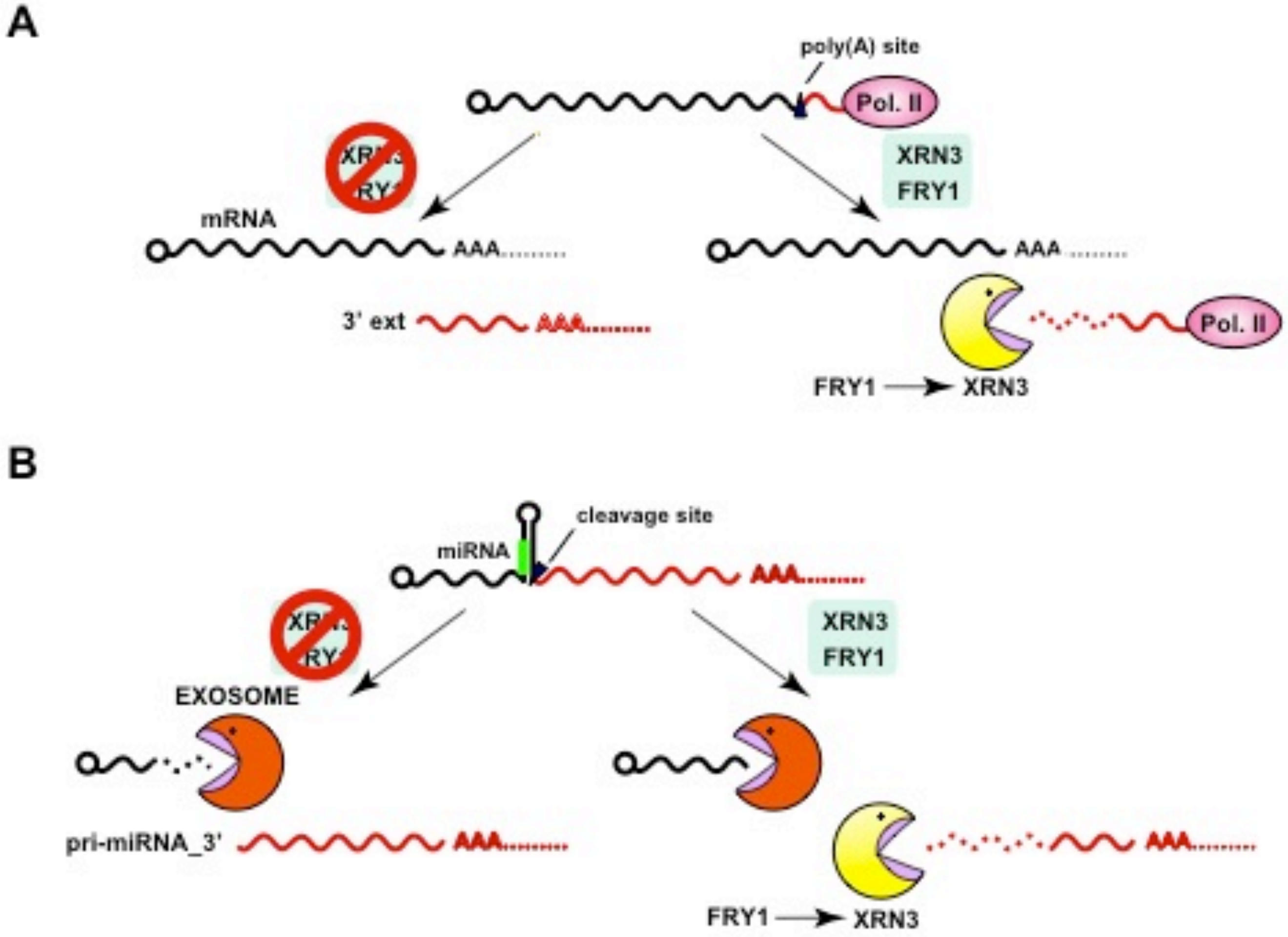

Figure S8 A proposed model for FRY1 and XRN3. (A) The 3' extension after cleavage at poly(A) sites during transcription is degraded in a 5'-to-3' direction by XRN3. (B) The 3' remnant of DCL1-mediated cleavage of pri-miRNA is degraded in a 5'-to-3' direction by XRN3.
